# Beyond Rehabilitation of Acuity, Ocular Alignment, and Binocularity in Infantile Strabismus

**DOI:** 10.3389/fnsys.2018.00029

**Published:** 2018-07-18

**Authors:** Chantal Milleret, Emmanuel Bui Quoc

**Affiliations:** ^1^Center for Interdisciplinary Research in Biology, Centre National de la Recherche Scientifique, College de France, INSERM, PSL Research University, Paris, France; ^2^Department of Ophthalmology, Robert Debré University Hospital, Assistance Publique - Hôpitaux de Paris Paris, France

**Keywords:** infantile strabismus, extending rehabilitation, cortical plasticity, interdisciplinary approach

## Abstract

Infantile strabismus impairs the perception of all attributes of the visual scene. High spatial frequency components are no longer visible, leading to amblyopia. Binocularity is altered, leading to the loss of stereopsis. Spatial perception is impaired as well as detection of vertical orientation, the fastest movements, directions of movement, the highest contrasts and colors. Infantile strabismus also affects other vision-dependent processes such as control of postural stability. But presently, rehabilitative therapies for infantile strabismus by ophthalmologists, orthoptists and optometrists are restricted to preventing or curing amblyopia of the deviated eye, aligning the eyes and, whenever possible, preserving or restoring binocular vision during the critical period of development, i.e., before ~10 years of age. All the other impairments are thus ignored; whether they may recover after strabismus treatment even remains unknown. We argue here that medical and paramedical professionals may extend their present treatments of the perceptual losses associated with infantile strabismus. This hypothesis is based on findings from fundamental research on visual system organization of higher mammals in particular at the cortical level. In strabismic subjects (as in normal-seeing ones), information about all of the visual attributes *converge, interact* and are thus *inter-dependent* at multiple levels of encoding ranging from the single neuron to neuronal assemblies in visual cortex. Thus if the perception of one attribute is restored this may help to rehabilitate the perception of other attributes. Concomitantly, vision-dependent processes may also improve. This could occur spontaneously, but still should be assessed and validated. If not, medical and paramedical staff, in collaboration with neuroscientists, will have to break new ground in the field of therapies to help reorganize brain circuitry and promote more comprehensive functional recovery. Findings from fundamental research studies in both young and adult patients already support our hypothesis and are reviewed here. For example, presenting different contrasts to each eye of a strabismic patient during training sessions facilitates recovery of acuity in the amblyopic eye as well as of 3D perception. Recent data also demonstrate that visual recoveries in strabismic subjects improve postural stability. These findings form the basis for a roadmap for future research and clinical development to extend presently applied rehabilitative therapies for infantile strabismus.

## Introduction and overview

**Figure 1:**
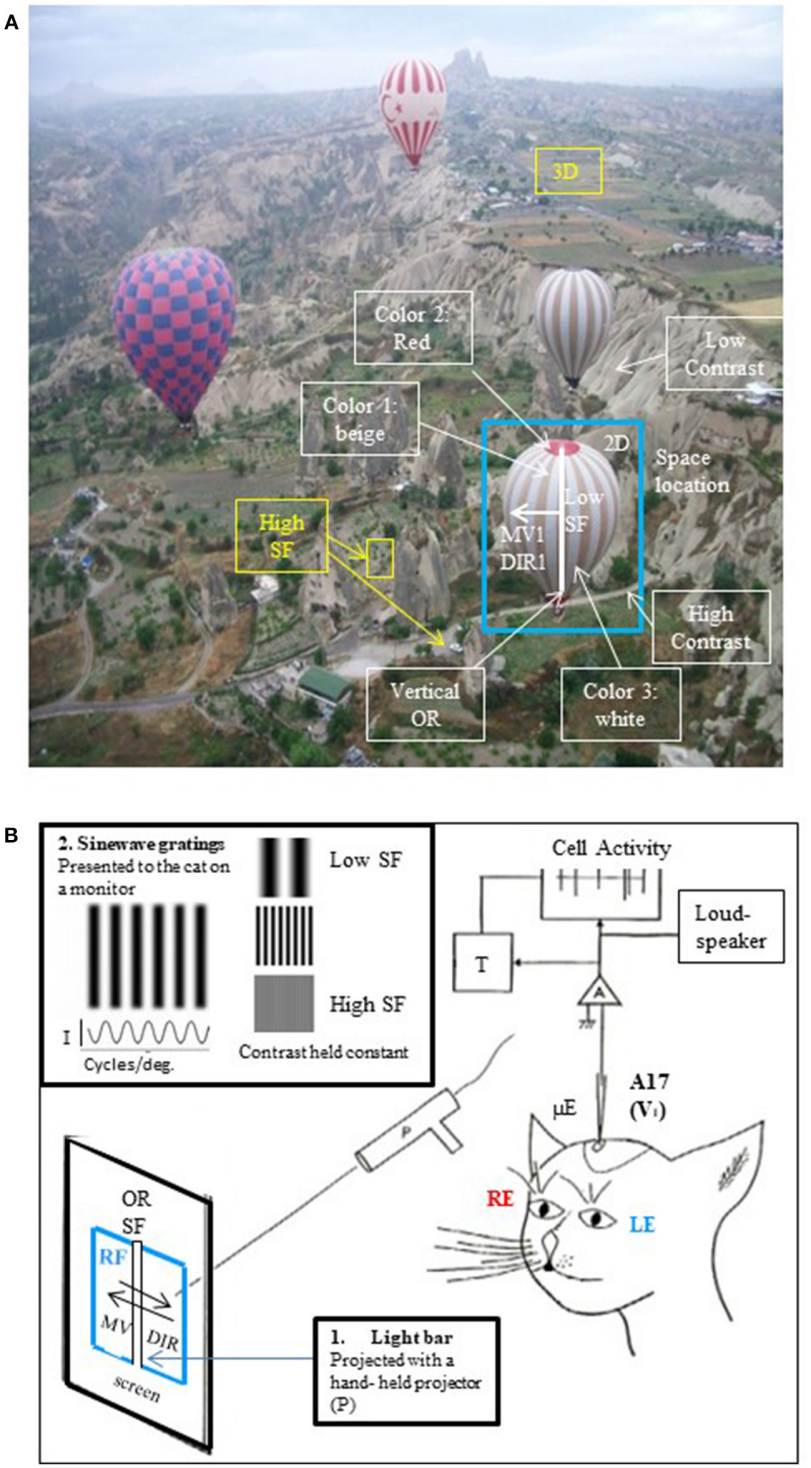
The visual scene and its different visual attributes. **(A)** A real visual scene and its various attributes. As a general rule, the visual scene may be decomposed into the so-called *visual attributes*, i.e., space locations, edge orientations (ORs), spatial frequencies (SFs, ranked as low to high ones corresponding respectively to gross to fine details in the visual scene), movement velocities (MVs), directions of movement (DIRs), 2D and 3D perception subtended by binocularity, contrasts and colors. To illustrate this here, a photograph taken from a hot-air balloon flying over Cappadocia in Turkey has been analyzed: the nearest hot-air balloon (selected in the blue rectangle) is located in front, on the right (= 3D and 2D localizations respectively). Its general orientation is vertical (vertical OR, white line). The balloon displays large vertical stripes of equal width (= low SF) which are alternatively beige and white (colors 1 and 3); in contrast, its top and its basket are red (color 2). This hot-air balloon is moving slowly (MV_1_) toward the left (DIR_1_). It is surrounding by 3 other hot-air balloons (= low SFs) which are located at various distances from one another (3D localization). At this altitude, the bushes and cars on the ground are small (= high SFs). They are however easy to distinguish from surroundings (high contrast). On the other hand, the mountain slope appears uniform despite the presence of some heterogeneous elements (low contrast). The SFs and contrast are indeed tightly linked (Campbell and Maffei, 1981; see text). After infantile strabismus, the perception of all of these attributes is altered. But only the losses of high SFs and 3D perception (in yellow) are presently treated by medical professionals. **(B)** Cue attributes used during experiments performed in animal models to explore functionally the primary visual cortex. In a real visual scene, the visual attributes are varied, scattered and mixed **(A)** rendering it difficult to understand how each one specifically activates V_1_ neurons. To solve this problem, Hubel and Wiesel (1962, 1965) positioned an anaesthetized, paralyzed cat in front of a screen oriented tangentially relative to the visual field. The screen projections were calibrated in degrees of the subject's visual angle. The area centralis (= foveas) and optic discs were also back-projected onto the screen to be able to determine the positions of the vertical and horizontal meridians onto the screen (Vakkur et al., 1963). Then the extracellular activity of single neurons was electrophysiologically recorded step by step (every 50 or 100 μm) in the different layers of the primary visual cortex (from layer I to layer VI) with a microelectrode (μE) oriented perpendicularly or obliquely with respect to the cortical surface. The spikes generated by each recorded neuron were amplified (A), continuously visible onto an oscilloscope (cell activity), transformed into impulsions (T) and transmitted to a “load-speaker.” This allowed online identification of spontaneous or visually evoked changes of the neurons' activity. For each recorded neuron, the *visual stimulus was a static or moving elongated (light or dark) bar manually projected (P) onto the tangent screen. Stimulus size and contrast were optimized by trial and error*. The left eye (LE) and the right eye (RE) were systematically stimulated separately. Of particular interest here was the innovation of the use of a bar as a visual stimulus permitting for the first time to identify each neuron's receptive field (RF) to which it is sensitive. Then, still using such bar, they systematically characterized the visual attributes (except for colors) that best activated each neuron in V_1_. These included the most effective orientation (OR), spatial frequency (SF), velocity and direction of movement (MV and DIR respectively) of the bar. The ocular dominance could also be determined by comparing visual responses to each eye individually. In more recent works, *sine-wave gratings* on a monitor placed in front of the animal were also used as visual stimuli for testing the respective attributes (e.g., Maffei et al., 1979; Albrecht et al., 1980; Albrecht and De Valois, 1981). An advantage of grating relative to bars is that the use of gratings also permitted to analyze precisely the neuronal responses to various SFs, which values could be determined with great precision (in cycles/deg; see inset at top left). I, luminance intensity.

The visual scene may be decomposed into what are referred to as *visual attributes*, i.e., image location, orientations (horizontal, vertical, oblique), spatial frequencies (ranked from low to high, corresponding to gross to fine details respectively), velocities/directions of movement, binocularity (subtending 2D and 3D perception), contrasts and colors (Figure 1A). In infantile strabismus, i.e., strabismus occurring during childhood, the perception of each of these visual attributes can be altered as well are vision-dependent processes such as postural stability. But presently rehabilitative therapies by ophthalmologists, orthoptists and optometrists are restricted to preventing or curing only a few perceptual deficits among these. In the interest of helping these medical and paramedical practitioners evolve these therapies, we (i.e., a fundamental researcher and an ophthalmologist) hypothesize here that rehabilitation after infantile strabismus should be extended further, and we develop arguments in favor of such hypothesis.

**Figure 2:**
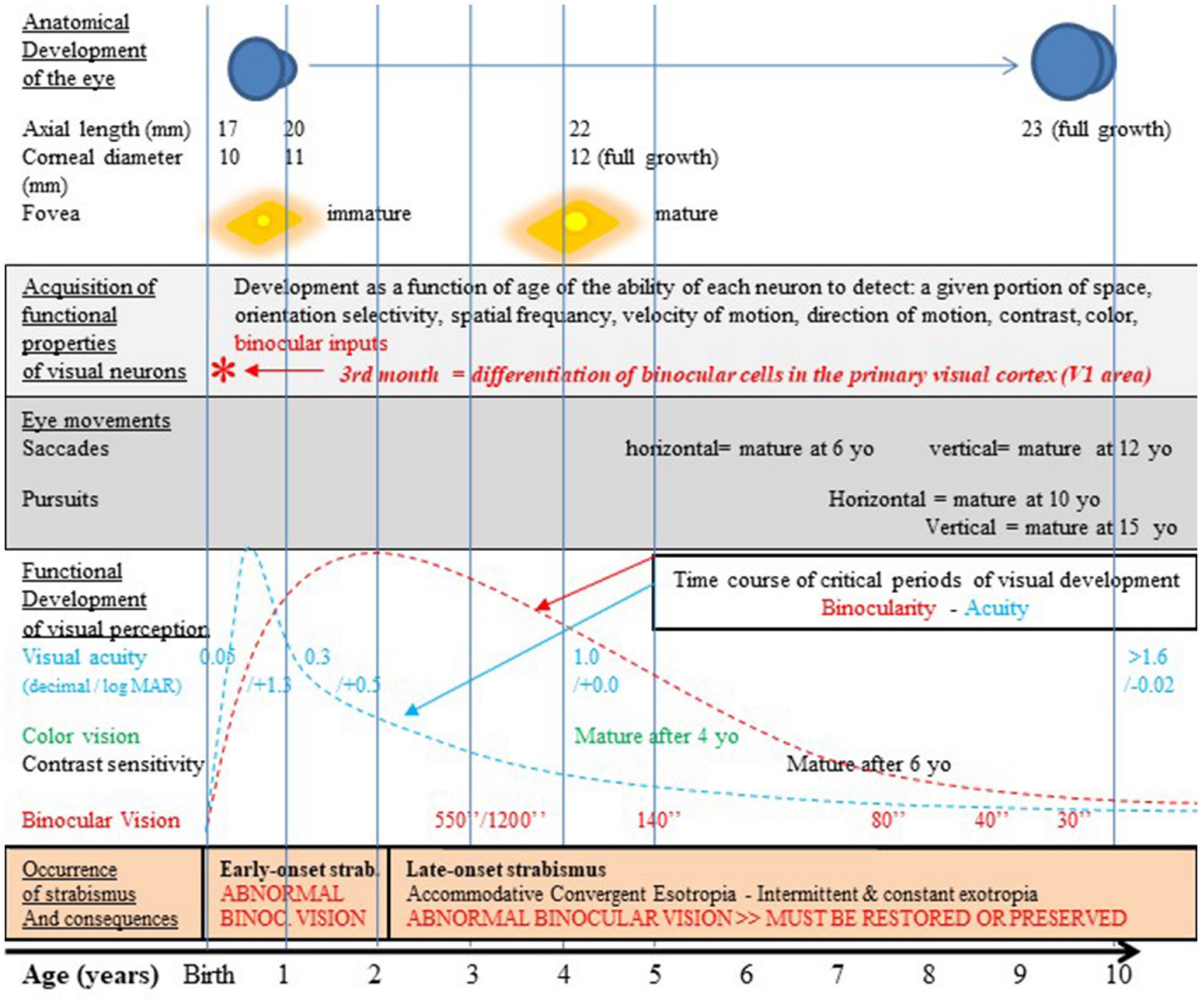
Normal visual development in humans after birth and strabismus onset timings. The development of the visual system occurs first pre-natal and continues post-natal until at least 10–12 years as illustrated here. It includes the growth of the eye, an increase of the corneal diameter and the progressive formation of numerous and organized connections between the eyes and the cortex. This latter process at least occurs in concert with functional changes which are strongly vision-dependent. Thus, the retina matures, in particular within the fovea. Neurons in sub-cortical and cortical structures also acquire progressively adult functional characteristics. Among the latter processes, neurons in V_1_ progressively acquire the capacity to be activated by stimuli of given positions in space and particular orientations, spatial frequencies, velocities and directions of movement, contrasts and colors. They also acquire binocular responses while they are initially mostly activated through the contralateral eye. In other words, cortical neurons progressively acquire specific selectivity for each visual attribute. Ocular movements such as saccades and pursuit also mature with age but not all at the same rate. Altogether, this leads to the development of visual perception including acuity, color vision, contrast sensitivity, binocular vision and 3D perception; this also leads to the development of space location, the ability to detect orientations and sensibility to movement (not indicated in the figure). All these processes occur during the so-called “critical period” of development which corresponds to a period of high plasticity with a peak during the first few post-natal years. Note that each visual attribute has its own critical period, with its own time course. For example, the critical period for acuity peaks at 3–6 PN months (blue curve; Epelbaum et al., 1993) while the one for binocular vision peaks later on at 1–3 years (red curve; Banks et al., 1975). Unfortunately, infantile strabismus (with early- or late-onset), which is associated to an abnormal post-natal visual experience, precisely occurs during these periods of very high plasticity (cf. the lowest part of the figure for details). The development of the anatomo-functional properties of the visual system and the development of visual perception of *all* the visual attributes may thus be greatly altered. But currently used treatments by medical and paramedical professionals and the strategy we propose in this paper may prevent, limit or eliminate such effects (cf. the text for further details). yo: years old; *, 3rd month: differentiation of binocular cells in the primary visual cortex. Reproduced from Figure 1 in Bui Quoc and Milleret (2014) with permission from *Frontiers in Integrative Neuroscience* and copyrights.

This is pertinent since infantile strabismus occurs in 2–3% of children worldwide, and is a rather complex pathology occurring at a key period in the development of the visual system. Recall that strabismus is characterized by the two eyes not aligning simultaneously under normal conditions. One or both of the eyes may be deviated medially, laterally, upwards or downwards from the forward resting gaze position. The orientation shift may be constant or intermittent. Accordingly, the origins of these problems may be multiple, i.e., peripheral or central, sensory or motor, genetic or epigenetic (Bui Quoc and Milleret, 2014). Whatever the type and origin of such misalignment of the eyes, the symptoms first appear in childhood (Figure 2). When they appear in the first 2 years this is referred to as “early” infantile strabismus (early onset strabismus; 10% of the cases) while when they appear later than this, it is considered as “late” infantile strabismus (90% of the cases). In all cases, unfortunately, this corresponds to the peak of sensitivity of the “critical period” (or “sensitive period”), i.e., the time window when visual processing circuits of the growing brain (which are the neural bases for visual perception) have elevated plasticity and show heightened responsiveness to environmental influences (Hubel and Wiesel, 1970). In humans, considering together the processing of *all* of the diverse visual attributes, this period begins globally soon after birth, peaks between 3 months and 3 years (depending on the attribute) and terminates at about 10–12 years of age (Banks et al., 1975; Leguire et al., 1991; Epelbaum et al., 1993; Keech and Kutschke, 1995; Lewis and Maurer, 2005).

Consequently, the development of visual perception is altered in cases of infantile strabismus. The earlier the strabismus is the more important the visual perceptive alterations are. The development of perception of *high spatial frequency* components of images is severely affected because of the mismatch of information coming from the 2 eyes, which can lead to amblyopia, and hence to a loss in visual acuity. The development of *binocularity* and the resulting *3D visual perception* might also be altered. *But it is less known that the development of perception of all the other attributes of the visual scene is also altered, including perception of image position, orientation, velocities/directions of movements, contrasts* and *colors* (cf. Figure 1A). In other words, infantile strabismus leads to a general alteration of visual perception (Ho and Giaschi, 2006, 2009; Davis et al., 2008; Thompson et al., 2008; Husk et al., 2012; Husk and Hess, 2013; Li et al., 2015; Meier et al., 2016; cf. also Milleret, 1994; Kiorpes and McKee, 1999; Ho et al., 2006; Kiorpes, 2006; Hamm et al., 2014; for reviews). Visually-dependent processes are also affected in infantile strabismus. This is well illustrated when considering *postural stability*. Visionary medical practitioners were the first to detect this (Marucchi, 1987; Marucchi and Gagey, 1987) and it has been confirmed more recently by researchers in collaboration with medical and paramedical practitioners (Lions et al., 2014; Ezane et al., 2015).

Thus, infantile strabismus must be treated comprehensively. Otherwise, the above-mentioned losses may persist systematically. A three-step program is presently applied for this during the first post-natal years (when plasticity of the visual cortex is maximal) by ophthalmologists assisted by orthoptists and optometrists. This can eliminate, or at least limit, the negative consequences of infantile strabismus and to restore to normal impaired functions as much as possible. But unfortunately only a few visual attributes are treated in this program and their restoration is not always possible. First, optic corrections of refractive and/or accommodative errors of both eyes are performed. Then amblyopia of the “lazy” (deviated) eye is eliminated (or reduced or prevented) through occlusion of the “best” (non-deviated) eye. This helps restore the acuity balance between the two eyes as much as possible. Finally, the visual axes of the eyes can be realigned through surgery of the extraocular muscles and their tendons. This can facilitate binocular vision (and thus stereopsis). Gaze symmetry is also desirable for “esthetic” reasons. These goals are achieved in many but, unfortunately, not all, cases. This is because the medical and paramedical professionals have to deal with several difficult problems. First, as indicated above, infantile strabismus may have different origins and there are several types, with sometimes very complex combinations of symptoms. Second, each eye muscle does not work in isolation: rather the six extraocular muscles of each eye work in coordination with the others and with those of the other eye; thus they interact with one another through biomechanical and/or proprioceptive and/or brainstem and/or cortical mechanisms. A consequence of this is that when one or several extraocular muscle(s) is (are) operated, there is some impact on the others and this is not always predictable. Third, the motor activity of these extraocular muscles is under central influences that are not completely manageable by medical and paramedical professionals. Fourth, while rehabilitation of perceptual losses such as binocular vision is possible, this is strongly dependent on the timing of the occurrence of infantile strabismus (early vs. late onset). Thus, early onset infantile strabismus is, in general, considered to completely prevent the development of binocular vision and thus the development of stereoscopic vision. This is because in these cases the neuronal networks underlying binocularity have not yet developed in the brain before strabismus onset and may not develop later on (cf. Figure 2). Thus, whatever the post-natal age, neither eye surgery nor intramuscular injection of botulinum toxin may be effective (cf. Klainguti, 2005; but see Banks et al., 1975 who reported development of some binocularity in subjects with “early” onset infantile strabismus with very early corrective surgery). As a consequence, in most, if not all cases, early onset infantile strabismus patients will be limited to monocular vision for the rest of their life. In contrast, in cases of late onset infantile strabismus, the neuronal connectivity underlying binocular vision has had time to develop during the critical period before strabismus occurs. In effect, even if their vision is functionally altered by the strabismus, these patients can still recover stereopsis, provided however that the strabismus was properly managed.

Regrettably, nowadays the other visual perceptive losses mentioned above, including image localization, orientation discrimination, detection of velocities/direction of movement, contrasts, colors and the postural losses, are not taken into account in the rehabilitative therapy of infantile strabismics. Yet these losses are no less important than those that are currently treated. Beyond the tendency of medical and paramedical professionals to focus on monocular visual acuity and binocular vision, this neglect of other perceptual symptoms results from at least three other reasons. The first is *the existence of some unavoidable limitations in the brain function that prevent any rehabilitation, whatever the medical action*. For example, as evoked above, it is generally considered impossible to establish binocular vision and thus depth perception in a patient with *early* infantile strabismus. The second reason is our *lack of knowledge*. For example, perception of each attribute of the visual scene does not mature at the same age although their respective developmental timelines display clear overlaps (Bui Quoc and Milleret, 2014; Figure 2). Experimental data from higher mammals (cats, monkeys) and observations in humans have shown that each visual attribute has also its own critical period with its own time course, although they have not yet all been established (Wiesel and Hubel, 1963; Berman and Daw, 1977; Daw et al., 1978; Harwerth et al., 1986; Wang et al., 2010; see also Daw, 1998, 2009; Kiorpes, 2015; for reviews). In humans, at least to our knowledge, the time courses of only two critical periods are indeed presently known precisely. Both the critical periods for the development of human binocular vision and the one for visual acuity start very soon after birth and end rather late at ~10–12 years of age. But the former critical period peaks between 1 and 3 years of age (Banks et al., 1975) while the later has been reported to peak at ~2 post-natal years (Epelbaum et al., 1993). Note that in the latter paper the age when plasticity is reported as maximal is however likely imprecise because of difficulties in testing visual perception in infants under 2 years of age. Based on other methods, including clinical practice, this peak for visual acuity more likely occurs earlier at ~3–6 post-natal months (Leguire et al., 1991), thus earlier than the peak for binocularity. The third reason that perceptual symptoms resulting from infantile strabismus are neglected is *the lack of therapeutically proven methods to rehabilitate perception of visual attributes* other than high spatial frequencies (i.e., acuity) and binocular vision. As a consequence, overall, rehabilitation after infantile strabismus is presently rather limited.

Here, the main goal is thus to explore possibly more comprehensive approaches and whether solutions may be proposed to circumvent the current limitations. The final aim is to motivate and inspire new strategies to rehabilitate impaired perception of *all* (or almost all) of the visual attributes and facilitate recovered or at least improved function in “visuo-dependent” processes such as those which subtend postural stability. Our hypothesis is that this might be possible because of the organization and the functioning of the *visual cortex* which are overall governed by the same principles in subjects with normal vision and patients with infantile strabismus. One underlying principle is the *convergence* of information about the different attributes of the visual scene, in particular at the cortical levels (area V_1_ and beyond), where global visual perception is elaborated. A second resulting principle is that of *interactions* and of *interdependency* of the various attributes of the visual scene during the elaboration of visual perception. Note that the visual system also has a substantial *impact* on the functioning of other “vision-dependent” systems. Each of these principles is developed below. Also of interest here, considering the extensive adaptative potential of the CNS, is how these principles may apply at different stages of development, including adulthood. The plasticity of the visual cortex is indeed maximal during the post-natal critical period, from birth to 10–12 years of age (as mentioned above) but some forms of plasticity still persist in adulthood (Milleret and Buser, 1984, 1993; Watroba et al., 2001; Baroncelli et al., 2011). After the detailed presentation of each of these principles, to support our hypothesis, a few examples will be provided for illustration. These will show the application of such principles and how they can guide new approaches through an inter-disciplinary approach to extending current therapies for rehabilitation of perception to multiple visual attributes and vision-dependent processes after infantile strabismus.

## Principle of convergence in visual cortex

### Convergence in primary visual cortex

In higher mammals with frontal normal vision, the different attributes of the visual scene are first processed in parallel (thus separately) within the primary visual pathway which includes the retina, the dorsal lateral geniculate nucleus located in the thalamus and the primary visual cortex (area V_1_, or A17).

The first evidence that parallel pathways in the mammalian visual system process different visual attributes came from electrophysiological recordings in the retina of the cat. Enroth-Cugell and Robson (1966) showed that a group of cells called *X-ganglion cells* respond to contrast and spatial frequency of an image. Others called *Y-ganglion cells* respond preferentially to moving stimuli. A third group of *W-ganglion cells* show still different (and very heterogeneous) functional characteristics (Wässle and Boycott, 1991 for review). Interestingly, these functionally distinct X, Y, and W ganglions cells correspond respectively to morphologically distinct β, α, and γ retinal ganglion cells whose proportional distributions are 45, 5, and 60% (Stone, 1983). These three classes of ganglion cells are at the origin of distinct pathways projecting differently through the dorsal lateral geniculate nucleus to the primary visual cortex which includes, in the cat, the three areas A17, 18, and A19 (cf. Payne and Peters, 2002 for details).

**Figure 3:**
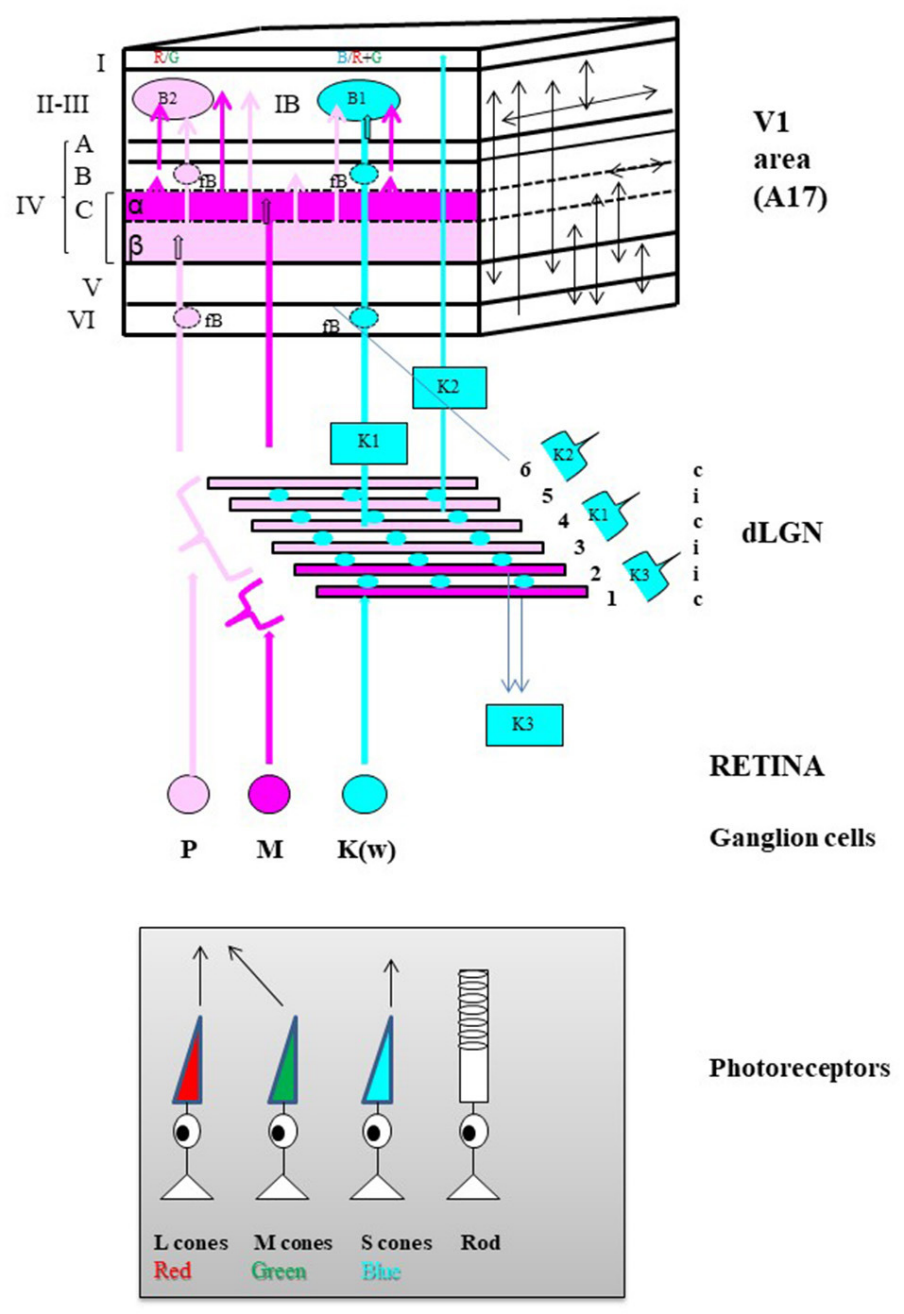
General anatomo-functional organization of the primary visual pathway in primates. Main organization of the three “primary” parallel visual input channels. The primary visual pathway transmits ~90% of the retinal inputs to V_1_. It is classically divided into three channels running in parallel designated respectively as magnocellular (M), parvocellular (P) and koniocellular (K_(W)_) which each process information about different subsets of visual attributes, but all respecting retinotopy, i.e., with receptive fields relative to their position in the retina. The M channel (in Magenta) mainly processes information relevant to the perception of motion, originating almost exclusively from rods located in the peripheral retina while the P channel (in light Pink) processes information relevant to the perception of form and colors (Red [R] and Green [G] only), originating from the long-wavelength (L, in red below) and the middle-wavelength (M, in green) sensitive cones mostly located in the central retina. Finally, the K channel processes information relevant to only the perception of the color blue since it originates strictly from the short-wavelength (S, in blue) cones also mostly located in the central retina. Retinal processing develops some of the attribute characteristics of the respective channels which then leave the retina via axons of their M, P, and K_(W)_ retinal ganglion cells (GG) respectively. Note that the latter group of GGs is referred to a “K_(W)_” because it includes GG cells which have not a specific name in the primate but which are similar in physiology and connectivity to the bi-stratified GG cells belonging to the W group in the cat retina (Hendry and Reid, 2000). Then the GG cell types of each eye project bilaterally to dedicated layers in the thalamic dorsal lateral geniculate nucleus (dLGN). M type GG cells project to the 2 deepest dLGN layers numbered 1 and 2 in the Figure (Magnocellular layers, in Magenta) while the P cells project to the 4 superficial layers numbered 3–6 (Parvocellular layers, in light Pink). Note the illustrated distributions from the contralateral (c) and ipsilateral (i) eyes. In contrast, the K_(W)_ GG cells project to the inter-laminar regions of the dLGN as intercalated layers (Blue circles). Finally, the respective dLGN layers and inter-laminar regions have distinct projection patterns within V_1_ while still respecting retinotopy along the cortical surface. Of the six main cellular layers of V_1_ (in roman numerals), layer IV is the main target for geniculate inputs. It is composed of three different sub-layers: IV-A, IV-B, and IV-C with layer IV-C further sub-divided into sub-layers IV-C-α and IV-C-β. The M retino-geniculo-cortical channel terminates in layer IV-C-α while the P channel ends in layer IV-C-β. The main part of the K channel originates from the middle parts of the dLGN (indicated as “K1”) and terminates in patches within layers II and III called “blobs” (B1, in Blue). Note that layers II and III are usually combined (as layer II-III) because they are difficult to be dissociated whether functionally or on the basis of histological material; also blobs may be rendered visible in histological preparations reacted for the energy metabolism marker cytochrome oxidase (not shown). A smaller projection also originates from the dorsal parts of the dLGN (indicated as “K2”) and terminates in layer I. The final dLGN output pathway originates from the ventral parts of the dLGN (indicated as “K3”) and projects outside V_1_ to superior colliculus (Hendry and Reid, 2000). Numerous intracortical connections are then established both between the different cortical layers and within each cortical layer of V_1_ (schematized as arrows on the plane at the right). For example, both the M and P channels project to all the blobs B1 and B2 (B2, see below) as well as outside the blobs (IB, inter-blob zone) of layer II-III. A few important notes about the organization of color processing: (a) By receiving information from both the P and K channels, the B1 blobs are dedicated to Blue/Yellow (B/Y) opponency, with Y resulting from the combination of R and G; (b) In contrast, by receiving information from the P channel, the B2 blobs are dedicated specifically to R/G opponency. Thus there are two distinct processors of colors: B/Y and R/G; (c) B2 (R/G opponency) blobs are 3 times more numerous than B1 (B/Y) blobs; (d) Neurons of the same color opponency seem to form vertical columns from layer II-III to layer VI, including the faint blobs (fB) of layers IV-B and VI; (e) Cells in the same type of color opponency blob have short intra-blob connections, and display correlated activities; (f) Cells belonging to adjacent blobs of the same type (i.e., which process the same color opponency) display correlated activities through short to long horizontal connections (e.g., Livingstone and Hubel, 1984a,b; Ts'o and Gilbert, 1988 for further details).

An equivalent organization was then found in primates (including humans). Three channels referred to as the parvocellular (P), magnocellular (M), and koniocellular (K) channels respectively ensure the processing of the different visual attributes. They correspond to the X, Y, and W channels in the cat respectively. As illustrated in Figure 3, each channel also originates from a distinct set of retinal ganglion cells (M, P, and K respectively) which project in different manners to the dorsal lateral geniculate nucleus and then to V_1_ (corresponding to A17 only in primate). Livingstone and Hubel (1988) established that the P channel processes information relevant to the perception of form and color [red and green only, originating from the long-wavelength (“red”) and the middle-wavelength (“green”) cones], while the M channel processes information relevant to the perception of motion (originating mostly from the rods). Hendry and his collaborators then established that the K channel processes information relevant specifically to the perception of the color blue by originating strictly from the short-wavelength (“blue”) cones; this information is however associated to “red-green” information, i.e., “yellow” at the cortical level (Hendry and Yoshioka, 1994; see also Hendry and Reid, 2000 for review). Note that the organization of the above described color channels is directly related to the fact that the perception of color originates from a comparison between “red” vs. “green” and “blue” vs. “yellow.”

Nevertheless, whether in cat or primate, information processed through these different channels finally interact strongly in V_1_. Each indeed differentially projects across the six cortical layers (I–VI) of V_1_ but numerous intra-cortical connections extensively inter-link these different layers both vertically and horizontally (Figure 3; see also Payne and Peters, 2002). Thus, as developed below, *most neurons and most neuronal networks in V*_*1*_ *can each be activated by most visual attributes, leading us to introduce here the notion of “convergence.”*

#### Convergence at the level of single neurons

**Figure 4:**
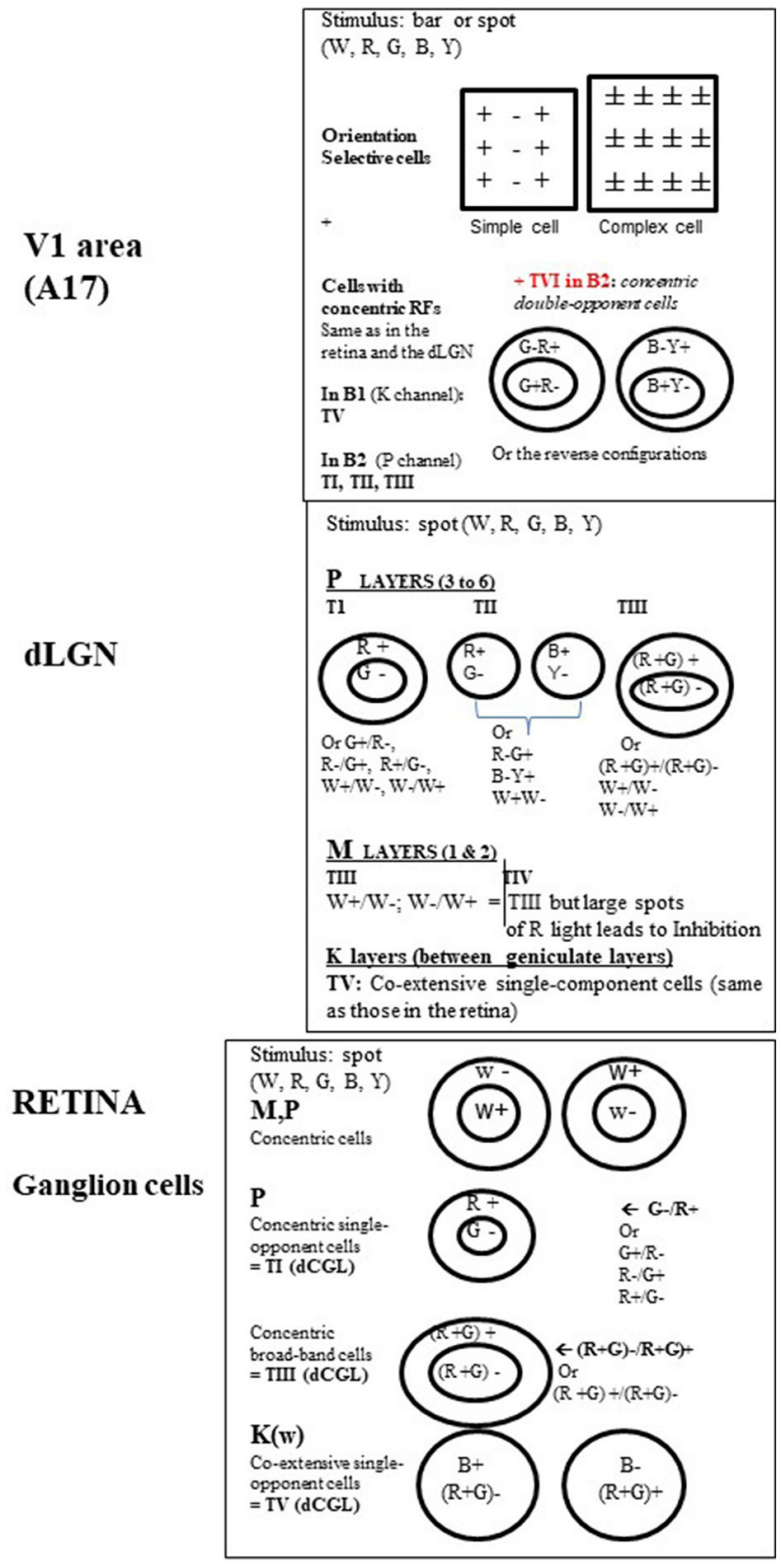
Organization of the visual receptive fields (RFs) from retina to V_1_ in the primate. **Retina**. In M, P, and K channels, the RFs of the ganglion cells (GGs) are all circular. This is shown using a stationary spot, the most effective stimulus at this level (Kuffler, 1953). In M and P channels, the GG RFs additionally respond to a light flashed “ON” at the center and “OFF” in the surround or display the reverse organization. In other words, they are of concentric center-surround opponent organization. But while M pathway GG cells only respond transiently to white (W) light (associated to motion detection), P pathway GG cells responses are sustained (i.e., last as long as the visual stimulus is present in the RF) and may be evoked by white (W), red (R) and green (G) light for both form and color detection. Two types of P cells have been distinguished while activated by R and G light: (a) the *concentric single-opponent cells* (P, 1st row) with for example a RF with center G- and a surround R+ (see other possible combinations at right in the Figure). This is center-surround spatial and chromatic opponency; b) the *concentric broad-band cells* (P, 2nd row) for which, for example, the RF includes R and G both in the center and the surround but with opposite actions. In the K channel (bottom row), the GG RFs do not display any concentric organization but display *co-extensive single opponent* responses, with opponent responses to blue (B) and Yellow (Y) light, the latter resulting from R and G association (cf. Schwartz et al., 2000). They are evidently also implicated in color detection but they are the least numerous of the GGs. Note that the RFs associated with color perception also occur in the dLGN and were labeled types I, III, and V (T_I_, T_III_, and T_V_ respectively) by Wiesel and Hubel (1966). For convenience, this same nomenclature is applied here for the retina. **dLGN**. While the retina and the dLGN thus share some RF types, some new ones emerge here as well. Again, RFs are all circular but not all are of concentric organization. *In the parvocellular layers (P)*, in addition to types T_I_ (the most frequent: 80%) and T_III_ ones, Wiesel and Hubel (1966) defined type T_II_. Contrasting with others, it exhibits opponent responses (W+/W- or R/G or B/Y) but no center-surround arrangement and thus have been designated as *single opponent cells. In the magnocellular layers (M*), beside type T_III_ with solely W+/W- opponent between the center of the surround of the RF, is one type of color-sensitive RFs labeled as T_IV_ RFs. It resembles T_III_ RFs but large spots of R light produce a dominant and long-lasting inhibition of activity (Inh.). Considering that this latter type is “broad-band” with respect to color detection, and that only few cells were concerned, the role of the M pathway in color perception is considered to be rather limited. Finally, only T_V_ RFs are in the inter-laminar *K zone of the dLGN* (Schwartz et al., 2000). On the basis of these characteristics*, both the GG cells and the dLGN neurons are considered primarily to detect spatial position, luminance and contrast. In addition, T*_*I*_ *and T*_*V*_ *cells are specifically considered as “performing a sort of calculation that is necessary to disambiguate wavelength and intensity” and as “building blocks for color vision” (Conway*, *2009**)*. Note however that a few cells in both the retina and dLGN are better activated by stimuli of a given orientation (OR) and/or a particular direction of movement (DIR) of a visual stimulus (e.g., Soodak et al., 1987; Tailby et al., 2010; see also Wei and Feller, 2011; Vaney et al., 2012), characteristics principally found at the cortical level (see below). This indicates that, in spite of the segregation M, P, and K at these sub-cortical levels, a few neurons may be already activated by different visual attributes. **V**_1_. In contrast with the retina and dLGN, *most RFs* in V_1_ are rectangular in shape, are orientation selective and are better activated by a moving W light bar of a given orientation. But stationary bars of a given orientation are also effective. Hubel and Wiesel (1962, 1965) first described these RFs as two main groups: *simple*, with alternating parallel regions responding respectively to ON and OFF light flashes (spot or bar), and *complex* which contrastingly display ON and OFF responses everywhere in their RFs. V_1_ *cells are also activated by the respective visual attributes without color responses (except a few C cells; cf*. Table 1). The remaining RF_S_ in V_1_ recall those of the retina and the dLGN, being circular with concentric organization and responses to W, R, G, B, and Y light (Livingstone and Hubel, 1984a; Ts'o and Gilbert, 1988). These include type T_I_ (*concentric single-opponent cells)*, T_II_ (*single opponent cells*), T_III_ (*concentric broad-band cells*), T_IV_ (*atypical concentric broad-band cells*) and likely also T_V_ ones (*co-extensive single-component cells*) RFs, with a specific distribution in the blobs B1 and B2 (cf. Figure 3). Another RF type found in B2 blobs are referred to here as Type VI (T_VI_). These display center-surround RFs but each portion of the RF may respond to two colors (R/G or B/Y) and the response to a given color is reverse in sign in the center and the surround (cf. figure, on the top at right). In contrast with the other RF types, T_VI_ responds poorly, or not at all, to white light of any form, or to diffuse light at any wavelength. The corresponding cells are called *concentric double-opponent cells*. Although not numerous (5–10% of V_1_ neurons), these cells are considered *to underlie perception of local color contrast and color constancy (but not of color itself) by comparing color signals across visual space* (e.g., Conway, 2009 for review). Thus they would contribute to perception of changes in the appearance of a color when contrasted with another one (for example, gray looks bluish if surrounded by yellow), and to make a color constant under very different viewing conditions. *Globally, V*_1_ *thus includes two distinct neuronal populations, those with rectangular RFs and others one with circular ones, with very different properties*. See Table 1 for further details.

In their 1981 Nobel prize winning work, Hubel and Wiesel (1962, 1965) first showed with extracellular electrophysiological recordings that single neurons in V_1_ can be activated by several visual attributes of the visual scene. To do this they projected a static or moving elongated (light or dark) bar of optimal size (in terms of length and width) and optimal contrast onto a screen facing the animal (cf. Figure 1B for details) and tested each eye successively. They demonstrated that most V_1_ neurons respond selectively to the following attributes:

- *A specific region of visual space*. This region was generally *rectangular* and is referred to as the neuron's *receptive field* (RF in Figure 1B). Importantly, the neurons in V_1_ are organized by *retinotopy*, that is, along the surface of the cortex they are arranged according to the positions of their receptive fields in the retina. Note that a single neuron can have two overlapping receptive fields stimulated by the respective eyes.
- *A preferred stimulus orientation* which can be horizontal, vertical or oblique. This was shown by comparing responses to *static* bars projected ON and OFF onto the screen within the receptive field at various orientations. Two main types of orientation selective cells (with rectangular receptive fields) were identified in V_1_ (cf. Figure 4): (i) *simple cells* whose receptive fields displayed adjacent parallel regions responding respectively to ON and OFF visual stimulations. The orientation of these parallel regions defined the most effective orientation of the visual stimulus to activate the cell since the ON zones and the OFF ones are antagonistic; (ii) *complex cells* which displayed ON and OFF responses throughout their receptive fields. The orientation selectivity of these cells (whether simple or complex) was confirmed (and appeared even more clearly) by projecting the image of a *moving* bar of various orientations. Note that orientations at slight angular deviations from the most effective orientation also activated the recorded neuron but with decreasing efficacy which progressively diminished to zero when a certain angle was reached. This allowed them to define an angular range of orientation selectivity for each cell.
- *A certain range of movement velocities*. This was established by moving the image of the bar on the screen in a direction perpendicular to the orientation preferred by the neuron (Figure 1B). Orban et al. (1981) further demonstrated that neurons in cat primary visual cortex could be ranked into four main classes according to their movement response characteristics: “low pass” and “high pass” neurons responding respectively to only low or high speeds (up to 700°/s!), while “tuned” cells respond selectively to certain intermediate speeds and the “broad-band” cells respond to all speeds of movement of the visual stimulus.
- *One or two opposite directions of movement*. This was also established by moving the image of the bar perpendicular to the preferred orientation.
- *The highest luminance contrast*. This was assessed by studying the activity of each neuron in V_1_ while increasing the contrast between the luminance of the bar and the screen.

In addition, Hubel and Wiesel demonstrated that most (~90%) neurons in cat V_1_ (A17) can be activated through both eyes, which strongly contrasts with geniculate neurons which are almost all activated by one eye (cf. Figure 3). In other words, *most neurons in V*_*1*_ *are normally binocular*. This is very important here since pathways conveying all visual attributes converge from each eye onto virtually almost each cortical neuron. This thus leads to aberrant processing in cases of infantile strabismus because the bilateral ocular inputs do not correspond to the same part of the visual field and the precision of this convergence is fundamental for 3D perception (further discussed below).

These seminal studies have been confirmed and built upon by other observations, in various mammalian species including primates (e.g., Zeki, 1983; Hubel and Wiesel, 1988). Thus, for example, it has also been shown that:

- Most neurons in V_1_ *are specifically activated by a larger portion of visual space* than that described initially by Hubel and Wiesel (1962, 1965). With intracellular recordings (instead of extracellular ones), Bringuier et al. (1999) demonstrated that the visually evoked “synaptic integration field” of each neuron is in fact 6-8 times larger than previously thought. In other words, in addition to the “classical” receptive field established on the basis of spike activity, they found an additional surrounding zone in which changes of membrane potential of the neuron could be detected, but without evoking action potentials. This subthreshold activity was characterized by responses to flashed stimuli which decreased in strength and increased in latency at increasing distance from the “classical” receptive field (see also Frégnac et al., 1996 for review). Importantly in the present context, spikes from neurons recorded in V_1_ of cat have been detected in this same zone which is 6–8 times larger than the “classical” receptive field only a few weeks after monocular strabismus or monocular occlusion is induced in adulthood (Milleret and Buser, 1993; Watroba et al., 2001; Milleret et al., 2005). A likely explanation is that the “normal” sub-threshold activity may grow to become spiking activity as a compensatory consequence of the pathological viewing conditions. Supporting this, the same receptive field expansion was observed in cat V_1_ after partial deafferentation of the retino-geniculo-cortical pathways through either optic chiasm section (Milleret and Buser, 1984) or retinal lesions (Gilbert et al., 1996; Abe et al., 2015).
- Most neurons in V_1_ *are activated preferentially by a specific spatial frequency*. This was demonstrated by combining extracellular recordings in V_1_ and the use of sine-wave gratings of various spatial frequencies as visual stimuli instead of a single bar (e.g., Maffei et al., 1979; Albrecht et al., 1980; Albrecht and De Valois, 1981). The sine-wave gratings were indeed pertinent since they included light and dark stripes which intensity changes gradually, in a sinusoidal fashion and their spatial frequency can thus be expressed easily and with precision as a number of cycles per degree of visual angle (cf. Figure 1B). Similar to other attributes such as orientation and velocity/direction of movement (see above), spatial frequencies near the preferred spatial frequency were less effective while very different spatial frequencies were ineffective.
- Most neurons in V_1_ display *binocular disparity*. This attribute is characteristic of binocularly activated neurons only, i.e., those neurons which display one receptive field for each eye (these receptive fields are located in the binocular visual field). The spatial overlap of the two receptive fields may be exact and thus binocular disparity would be nul. But, most often, receptive fields of a pair are rather separated very slightly (< 1°), referred to as *position disparity* (which results from the *retinal disparity* of the image). Without going into detail, depending on the relative spatial position of the two receptive fields, this disparity may be *crossed* or *uncrossed*, depending on whether the target is located in front of or behind the fixation plane. This is also fundamental here since it contributes to elaborate depth perception at the cortical level (Ohzawa et al., 1990; DeAngelis et al., 1991; Trotter et al., 1992; Poggio, 1995; Anzai et al., 1997; Trotter and Celebrini, 1999; Durand et al., 2007; see also Poggio and Poggio, 1984; Trotter et al., 2004 for review).

However not *all* of the visual attributes converge onto *all* neurons of V_1_. Investigations performed in primates, in particular concerning the neural bases for color perception, lead to the conclusion that information about *most* visual attributes converges onto *most* neurons in V_1_ while the remaining neurons receive fewer inputs with diverse attributes. In other words, there are not one but rather two neuronal populations in V_1_ (we shall call them Pop 1 and Pop 2 respectively) which differ both by their size and their degree of convergence.

These two neuronal populations are quite distinguishable when synthesizing, for example, data from Livingstone and Hubel (1984a) and those from Ts'o and Gilbert (1988), who precisely defined the responses to the different visual attributes (including color) of neurons recorded systematically within the different cortical layers of V_1_ in foveal and para-foveal regions of non-human primates (cf. Table 1). The largest neuronal population with the most convergence (Pop 1; panels in gray in Table 1) is found in the inter-blob zones (IB) of layer II–III, layer IV-B, and layer VI as well as in layer IV-C-α and layer V (cf. Figure 3 and legend for details). *It includes strictly neurons with rectangular receptive fields (simple or complex; cf. Figure* *4**) which are activated specifically by orientation and velocity/direction of movement and which are also mostly binocular*. However, these neurons are not selective for color (except in part IB of layer II-III, where a few complex cells have been found to be color selective). In contrast, the population with low convergence is smaller (Pop 2; panels without color in Table 1). It is restricted to the blobs of layers II-III, IV-B, and VI (cf. Figure 3 for details), layer IVC-β and a few cells in both layers V and VI. *Its neurons only have circular RFs which are almost exclusively activated by colors (Types T*_*I*_*, T*_*II*_*, T*_*III*_*, T*_*V*_*, and T*_*VI*_*; see Figure* *4* *for details) and they are mostly monocular*.

**Table 1:** Comparison of the degree of convergence of information about the various visual attributes of the visual scene onto neurons recorded at various depths in foveal and para-foveal regions of V_1_ of primate macaque monkey (adapted from Livingstone and Hubel, 1984a; Ts'o and Gilbert, 1988).

			**RF**	**OR**	**MV**	**DIR**	**OD**	**Color**
**I**		Not any cell responding to visual stimuli						
**II–III**	**Blobs**		Circular	ε Only very few cells	+ some cells	– Binocular but strongly monocular bias	++	+++ T_VI_>T_III_>T_II__+_ T_V_
	IB		Rectangular	+++ C cells >>> S cells	+++	–	+++ Binocular	+ Few C cells are color selective (R>G>Y>B)
**IV**	**A**		+	Only few cells—Very thin cortical layer—Difficult to explore				
	**B**	**Blobs** (faint)	Circular	–	–	–	+ Only few cells (classes 3, 4, 5)	+ But not defined
		**IB**	Rectangular	+ Mostly S cells	+	+++ 2/3 of the cells	+ ½ monocular ½ binocular but with a strong preference for one eye	–
	**C**	α	Rectangular	++ Mostly S cells	+	–	+ Monocular only	–
		β	Circular	–	–	–	+ Monocular only	+++ T_III_ > T_I_
**V**	Most cells		Rectangular	++ (S, C?)	++	–	+ Binocular without strong eye preference	–
	**Only few cells**		Circular	–	–	–	–	+ T_VI_
**VI**	**Blobs (faint)** Few cells only		Circular	+	–	–	?	+ Few T_III_
	**IB**		Rectangular	++	++ Mostly C cells	++	++ Binocular	–

*I–VI, cellular layers of V_1_ from the most superficial to the deepest one; Blobs, mostly apparent in layer II–III but also slightly visible in layers IV-B and VI (Faint blobs); IB, inter-blob zone (cf. Figure 3 for details). Visual attributes considered here: RF, receptive field shape; OR, edge orientation; MV, movement velocity; DIR, direction of movement; OD, ocular dominance and color (Recall that cells selective for orientation OR are also selective for spatial frequency SF, not shown here). Analyzing the Table reveals that V_1_ neurons may be ranked into two quite different populations. The first one (Pop 1), including strictly simple (S) and complex (C) cells with rectangular RFs, i.e., the most common cells in V_1_, receives the greatest convergence of the various visual attributes information (in gray colored areas). They are distributed in IB of layers II-III, IV-B, and VI as well as in layers IV-C-α and V (in gray). By contrast the second one (Pop 2), located in blobs of layers II-III, IV-B, and VI as well as in layers IV-C-ß and V (in non-colored areas), displays the smallest size and the smallest convergence. It indeed mainly includes monocularly activated cells with circular RFs of type T_I_, T_II_, T_III_, T_V_, and T_VI_ cells underlying color perception (cf. definition and specific locations in Figure 4)*.

Considering the distribution of the M, P, and K channels in V_1_ (cf. Figure 3), the greatest convergence of the different visual attributes occurs in neurons activated through the M and/or the P channels (with the P channel being implicated in form perception). In contrast, the least convergence occurs in neurons mostly implicated in color perception, activated through the P and/or the K channels, which process respectively the antagonisms Red/Green and Blue/Yellow (cf. Figure 3).

*In cases of infantile strabismus*, as established again experimentally through electrophysiological recordings in V_1_ of animals (cats and non-human primates), both the Pop 1 and Pop 2 neuronal populations in V_1_ still exist and remain different. The general organization of the visual system is indeed sustained. But responses to each visual attribute are altered in both populations (*Pop 1*: Chino et al., 1983; Kiorpes et al., 1998; Milleret and Houzel, 2001; Schmidt et al., 2004; Milleret et al., 2005; Watanabe et al., 2005; Bui Quoc et al., 2012; see also Von Noorden, 1978; Milleret, 1994; Wong, 2012 for reviews; *Pop 2*: Koçak-Altintas et al., 2000; Davis et al., 2008; Rajavi et al., 2015). Thus, in the Pop 1 neuronal population, each neuron still responds selectively to a specific region of visual space but some of them display larger receptive fields than in normal vision. About half of the neurons are still simple or complex (thus orientation selective) but neurons selectively activated by the vertical orientation may be rare or absent (in particular in case of infantile strabismus in the horizontal plane); the other half of neurons are not orientation selective. Also, neurons still respond best to a given spatial frequency but the low spatial frequencies become the most effective at the expense of high spatial frequencies; spatial frequency specificity is also generally lower than in normal vision (responding to a wider range of spatial frequencies). *Altogether, the large receptive fields, the poor orientation selectivity and the poor spatial frequency selectivity observed in Pop 1 are the neural bases for amblyopia*. Furthermore, most neurons now appear as monocularly driven through one eye or the other; only few binocular neurons may still be found. This is because, although still present, interactions between both eyes are altered. Without going into detail, this underlies the “interocular suppression” which helps avoid diplopia (Chino et al., 1994; Sengpiel et al., 1994, 2006; Sengpiel and Blakemore, 1996; Smith et al., 1997; discussed below). Binocular disparities are consequently completely abnormal because of the lack of binocularity and the inter-ocular suppression. *The loss in binocularity and the impairment of binocular disparity are the neural bases for the poor (or even non-existent) depth perception*. In addition, while neurons in strabismics still respond to a specific range of velocities of movement, the effective velocities of movement are slower than normal (meaning that the effective temporal frequencies are lower than normal), in particular in the periphery of the visual field. Although neurons remain selective for one or two directions of movement, the average directional bias of responses is significantly reduced. Note however that although eye movements of human may be asymmetric in case of early onset infantile strabismus, no prevalence of responding to naso-temporal directions of stimulus movement was found in animals in case of convergent or divergent infantile strabismus. Finally, the sensitivity to contrast also decreases markedly, whichever eye is visually activated. *In the Pop 2 neuronal population*, neurons retain their ability to respond rather specifically to color signal originating from one eye despite the pathology. But their responses to color are lower and slower than normal whether the amblyopic or the fixating eye is visually stimulated. *Altogether, this indicates that the neural bases for the perception of all the visual attributes are altered after infantile strabismus*.

Experimental approaches in animals have also allowed to establish that in case of infantile strabismus: (a) the more the central vision is concerned, the more the impairment is marked; (b) the more the deviation of the eyes develops near the peak of the critical period of a given attribute, the more the impairment is substantial (e.g., ocular dominance: Yinon, 1978; see also Figure 2); (c) there is no discernible relation between the degree of alteration of the neural bases for visual perception and the amplitude of the angle of deviation of the ocular axes after infantile strabismus (Yinon, 1978; Kalil et al., 1984); (d) M, P and K channels are all affected; (e) Among the above reported abnormalities, some at least likely result from the maintenance of functional immaturity in V_1_ because of the misalignment of the eyes (for example large receptive fields, lack of orientation selectivity, poor ability to detect fast motions, etc…). Note that such observations were primarily made in experimental models of “early onset” strabismus in animals. This involves muscle surgery (in general by removing the external rectus) at an early age. Thus, it is rather a palsy that is created and it is not exactly similar to what occurs in human with innate infantile strabismus. But, the research community generally concurs that such data are applicable to humans with in particular an early onset strabismus, although the question of cause vs. effect must still be discussed (cf. Bui Quoc and Milleret, 2014 for details).

- Most neurons in V_1_ (belonging to the Pop 1 population) *integrate* information about most of the visual attributes, both in normally viewing and infantile strabismic human subjects. In other words, information about most attributes of the visual scene *converges* at the level of most single neurons in both viewing conditions.
- The remaining neurons in V_1_ (belonging to the Pop 2 population) *do not integrate as many types of attribute information as the Pop 1 population*, whether subjects have normal sight or are strabismic since they have *much less convergence* of information of the respective attributes (in most cases, color and information from one eye only).
- In both populations, however, proximity *between information from* different visual attributes during visual perception may unavoidably favor *interactions*, an aspect which also interests us here (further discussed below).

#### Convergence within the cortical neuronal networks

As a general rule, each neuron in V_1_ of higher mammals (such as cat and primate) functions as an integrated element of intracortical and/or interhemispheric neuronal networks which have both excitatory and inhibitory connections. This inspires the question of whether and how the *principle of convergence* described above might be extended to these neuronal networks. The following sections describe how this takes place both in subjects with normal vision and in infantile strabismus. What is possible because the neurons sharing the same properties are always preferentially interconnected whether considering single or several visual attribute(s).

##### Anatomical organization of the neural networks of primary visual cortex

Briefly, the neuronal networks in V_1_ of higher mammals with normal vision are composed of both vertical and horizontal cortical connections (cf. Figure 3 for a summary in the primate):

- *Vertical connections* form intra-cortical networks only. In primate, they originate mostly from layers IV-C-α and IV-C-β which receive projections from the M and P channels respectively. Both project principally to layer II-III. From there, connections are established with all the other cortical layers through various vertical intracortical connections (e.g., Mitzdorf and Singer, 1978; Livingstone and Hubel, 1984a,b; Mitzdorf, 1985; Bolz and Gilbert, 1986; see also Gilbert, 1983; Payne and Peters, 2002 for reviews).
- *Horizontal connections* may be short or long-range intra-laminar connections and thus may form intra- or inter-hemispheric networks. They mostly originate from layer II-III although they may be present in all layers of V_1_ except apparently in layer I, and these are often reciprocal (e.g., Gilbert and Wiesel, 1983; Rockland and Lund, 1983; Houzel et al., 1994; Buzás et al., 2001; Rochefort et al., 2009 for details; see also Kisvárday, 2016 for review).

*Globally, this organization of the cortical networks is sustained after infantile strabismus*. But it includes abnormalities because the retino-geniculo-cortical pathway has developed some abnormalities first (Garraghty et al., 1989; Löwel, 1994; Li et al., 2011; Duan et al., 2015). Thus, for example, intracortical horizontal connections in cat primary visual cortex are modified (Schmidt and Löwel, 2008; see also below). Long-range interhemispheric callosal connections also develop asymmetrically between the hemispheres instead of symmetrically as in normal vision because of the stabilization of some juvenile callosal projections within the hemisphere ipsilateral to the deviated eye during post-natal development, projections which are normally eliminated (Innocenti and Frost, 1979; Lund and Mitchell, 1979; Bui Quoc et al., 2012). Of interest here, the above described data about callosal connections have been established in cats with early induced unilateral convergent strabismus. But similar data have been found recently in humans with spontaneous infantile strabismus (Ten Tusscher et al., 2018).

- Neurons in V_1_ are thus anatomically interconnected and form more or less extended neuronal networks both within and between the hemispheres. This holds true in subjects with normal vision or with infantile strabismus. This indicates that neuronal networks in both viewing conditions are organized in such a way that they may subtend the functional convergence which interests us here and this occurs within the whole extent of V_1_.

##### Functional organization of the neural networks in the primary visual cortex

The *principle of convergence* also applies to the functioning neuronal networks in V_1_ of higher mammals. This is demonstrated here by first presenting the functional organization of the cortical neuronal networks implicated in the perception of a single attribute and then those underlying the perception of various attributes appearing simultaneously in the visual scene.

Functional organization of the neural networks implicated in the visual perception of one given attribute

**Figure 5:**
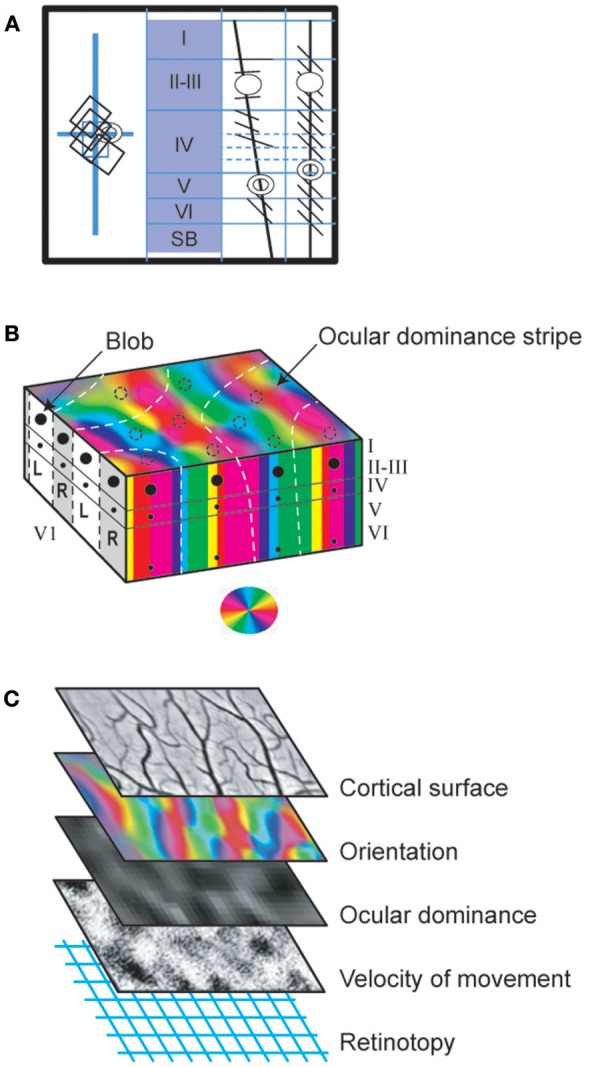
Functional organization of V_1_ neurons. **(A)** Columnar organization (demonstrated first by Hubel and Wiesel, 1963a with the protocol of Figure 1B). The visual responses evoked by neurons recorded in the respective oblique (3rd panel) or vertical (4th panel) electrode tracks were reconstructed from histological preparations, using as references the electrolytic lesions (represented as circles) applied through the microelectrodes at well determined depths at the end of each track. When the electrode penetration was vertical, thus perpendicular to the cortical surface, the neurons recorded in succession displayed overlapping receptive fields, i.e., were activated when overlapping and proximal portions of visual space were stimulated (rectangles in the left panel; vertical and horizontal blue lines correspond to the visual field meridians). Furthermore, all were activated by the same orientation (here diagonal) of the light bar (right two panels), thus demonstrating a *columnar organization*. In contrast, the oblique penetrations crossed various orientation columns and neurons successively lower in the same track were thus activated by different stimulus orientations rotating progressively from layer II-III through layer VI. Their receptive fields also gradually shift in the visual field (not shown). **(B)** From columns to cortical maps. Three distinct visual attributes (orientation, ocular dominance and color) serve here as examples to illustrate how columns of different attributes are organized to form maps in V_1_. In the block of V_1_ represented here, the vertical columns of neurons activated by the same *orientation* of the stimulus are represented by a given color according to the wheel shown below. Altogether they form a map which extends throughout the whole thickness of V_1_ and which is visible from the cortical surface. Note that, within this map, additionally, there are normally singularities where many different orientation columns abut, which are referred to as “pinwheels.” They are not visible in the present orientation map but they resemble the color wheel below. Concerning *ocular dominance*, L and R indicate columns activated by the left and right eyes respectively (represented in white and gray respectively at left of the panel). In the superior part of the block of V_1_, these ocular dominance columns are represented here as forming a map with “stripes” (represented this time as white dashed lines). Note that the ocular dominance map is normally most easily detected in layer IV, where the geniculo-cortical afferents terminate and are still separate for each eye. In the other cortical layers, cells are indeed mostly binocular preventing the establishment of ocular dominance map (see text). But an ocular dominance map as illustrated here can be detected from any cortical layer (except layer I) after an abnormal post-natal visual experience such as in infantile strabismus since most cells are monocularly activated whatever their location (see text). Concerning *color*, contrasting with the two previous examples, each column is discontinuous since it is formed by vertically aligned blobs in layer II-III as well as faint blobs in layers IVB and VI (represented here as black spots at left and at front of the figure; cf. also Figure 3 and legend for details). However, they form a color map visible from the brain surface with various imaging techniques as illustrated by the dashed circles. These diverse cortical maps have specific geometrical relationships. Thus, for example, the global ocular dominance map mostly crosses the global orientation map orthogonally. Also the blobs are mainly located in the center of the ocular dominance stripes. **(C)** Interlacing of cortical maps of different attributes in V_1_. One cortical map per visual attribute exists in V_1_ (except for contrast). Each may be visualized and characterized by optical imaging of intrinsic signals from the cortical surface following appropriate visual stimulations since each map extends through the V_1_ layers in columns. Some of these attribute maps are represented here: orientation and ocular dominance (where black and white indicate ipsi- and contralateral dominance) as global cortical maps while the movement velocity map shows only one velocity selectivity as black points. Note that each has a unique distribution pattern. Here it is as if they overlap each other. But in fact they are interlaced. This permits all permutations of the respective attributes to be represented in a column for each part of visual space (also see text). Reproduced from Figure 2.3 in C. (Milleret, 2017), with permission from *Elsevier Masson* and copyrights.

- Subjects with normal vision
  Those neurons of V_1_ which are activated by a particular attribute of the visual scene are first organized into *columns* oriented perpendicularly to the cortical surface and extending through all cortical layers (except layer I which is almost strictly composed of transversely connecting fibers). This constitutes a *modular organization* which is a general principle in the neocortex (cf. Mountcastle, 1997; for review). Hubel and Wiesel (1963a) again first showed this by using the same experimental protocol as described in Figure 1B. For example, they found orientation columns of neurons activated specifically by bars oriented at 45° (see Figure 5A). They also showed that such orientation columns are surrounded by other columns selective for other orientations. Hence columns selective for a particular value (such as 45°) of a particular attribute (such as orientation) are scattered and thus display a *discontinuous* distribution throughout V_1_. Note that the numbers of columns for the respective values of an attribute (for orientation, this would be the various angles) are equal, thus preventing bias favoring any particular orientation. However, in most cases, neighboring columns include neurons activated specifically by nearby values of the attribute (such as orientation angles). A progressive shift in preferred attribute values thus occurs with distance so that all 360° are covered by the network. This columnar organization applies to all other visual attributes except contrast (e.g., Hubel and Wiesel, 1962). Thus, for example, neurons recorded in a single vertical electrode track penetration in V_1_ may display similar ocular dominance. Of interest, all these columnar organizations in V_1_ are embedded in the retinotopic representation of visual space. Thus, neurons in each vertical electrode track include overlapping RFs (Figure 5A). But in contrast with the other visual attributes, the distribution of columns underlying the representation of visual space is *continuous* throughout V_1_. Note that such columnar organization are maintained after infantile strabismus in spite of the impairment of the functional properties of some neurons within these columns (e.g., Milleret and Houzel, 2001; Bui Quoc et al., 2012).
  Second, the neuronal columns activated by a given visual attribute form a specific functional *cortical map* (Figures 5B,C). Considering the characteristics of each column, not surprisingly, each of these maps extend both radially over layers II-III to VI and horizontally over the whole surface of V_1_. The orientation and ocular dominance cortical maps were first visualized *post-mortem* by combining radioactive tracer injections and monocular visual stimulation (*cat*: Löwel and Singer, 1987; *monkey*: Hubel and Wiesel, 1969; Hubel et al., 1978; LeVay et al., 1985). Then, from the 90's until recently, cortical maps have been preferentially characterized both in cats and monkeys by using optical imaging of intrinsic signals (e.g., Bonhoeffer and Grinvald, 1991). Imaging of activity from the surface of the cortex of experimental animals exposed to visual cues thus established *in vivo* the *global cortical maps* for each visual attribute (retinotopy, orientation, spatial frequency, velocity/direction of movement, ocular dominance etc.) by visualizing the distribution along the cortical surface of the tops of the active columns (appearing as “patches,” also called “domains”) (see Figure 5C). To demonstrate, for example, the global orientation map in a surface activity imaging experiment, visual stimuli with eight different orientations (covering 360°) are first presented separately to establish the corresponding “single cortical maps”; then all the “single condition maps,” each associated with a given color, are superimposed (Figures 5B,C). Note that, while the general organization is found in different individuals, the precise organization of the maps varies between individuals. The first global map that was visualized using such an imaging technique was of *ocular dominance* in primates. This was obtained by successive stimulations of each eye (Blasdel and Salama, 1986; Frostig et al., 1990; Ts'o et al., 1990; Grinvald et al., 1991). Many studies followed, establishing the global orientation cortical map, and showing its iso-orientation domains and singularities (“pinwheels”; cf. Figure 5B for illustration), with the respective orientation responses being arranged like the spokes of a wheel in which OR changes continuously around at the pinwheel center (Bonhoeffer and Grinvald, 1991, 1993a,b; Bonhoeffer et al., 1995; Maldonado et al., 1997; Shmuel and Grinvald, 2000; see also Schummers et al., 2002; Ohki et al., 2006). Up until recently, other functional global maps in V_1_ were characterized either with optical imaging or two-photon calcium imaging methods, including *retinotopy* (Bosking et al., 2000; Buzás et al., 2003; Schiessl and McLoughlin, 2003), *spatial frequency* (Issa et al., 2000; Nauhaus et al., 2012; Ribot et al., 2013, 2016), *direction of movement* (e.g., Bonhoeffer and Grinvald, 1993a; Shmuel and Grinvald, 1996; Kisvárday et al., 2000, 2001; Ohki et al., 2005), *color* (Livingstone and Hubel, 1984b; Lu and Roe, 2007, 2008), *binocular disparity* (Kara and Boyd, 2009) and *temporal frequency* (Yen et al., 2011). Not surprisingly, only the *retinotopic* global map was found to be organized *continuously* with “bands” of activity (corresponding to the various azimuths and elevations in the visual field) while the other global maps were found to be organized *discontinuously* (with “patches”). On the other hand, although neuronal activity in cortical maps of V_1_ clearly increases linearly with *contrast*, i.e., *luminance* (e.g., Lu and Roe, 2007), consistent with the absence of columns, no specific map has ever been found for this visual attribute: a contrast invariance was rather found over the whole extent of the cortex (cat: Carandini and Sengpiel, 2004, confirmed by Lu and Roe, 2007, in monkey). But a modular (thus discontinuous) representation of *luminance polarity* (ON or OFF) has been found recently in layer IV of V_1_ (Smith et al., 2015a; Vidyasagar and Eysel, 2015; Kremkow et al., 2016), which receives thalamic afferent inputs (cf. Figure 3 and Table 1). Importantly, because this ON-OFF organization originates from the clustering of ON and OFF thalamic afferents in V_1_, the authors propose that “all features of visual cortical topography, including orientation, direction of movement and retinal disparity, follow a common organizing principle that arranges thalamic axons with similar retinotopy and ON-OFF polarity in neighboring cortical regions” in V_1_. Note finally that sub-threshold facilitation and suppressive surround maps, in correlation with “active” zone and “silent” surrounding zones of receptive fields (see above) were also found in cat visual cortex (Toth et al., 1996; Vanni and Casanova, 2013; see also below in section Principle of Interactions and of Inter-Dependency of all the Attributes of the Visual Scene).
  In the healthy subject, these global V_1_ maps have several additional common important properties. Thus, the feed-forward retino-geniculo-cortical pathways as well as the intra-cortical and interhemispheric connections are organized congruently both anatomically and functionally, complementing one another. This ensures the convergence of information about each visual attribute in V_1_ in a coherent way (Bosking et al., 2000; Rochefort et al., 2007; Ribot et al., 2013). All the cortico-cortical connections (i.e., intra-cortical and interhemispheric ones) preferentially inter-link neurons with the same functional characteristics (e.g., Gilbert and Wiesel, 1989; Bosking et al., 1997; Rochefort et al., 2009) and thus ensure correlated activities within and between all the columns encoding for the same visual attribute in V_1_ of both hemispheres, for example those activated by the vertical orientation within the global orientation maps (Gray et al., 1989; Engel et al., 1991; Fries et al., 2002; see also Singer and Gray, 1995; Singer, 1999, 2013; Engel et al., 2001 for reviews). The mechanisms underlying functional architecture of V_1_ are so strong that the cortical representation of most visual attributes (thus the respective cortical maps) may emerge spontaneously, without any specific visual stimulation (Tsodyks et al., 1999; Kenet et al., 2003). Within a given global map, for example for orientation, activity changes within columns encoding for a certain orientation (for example vertical) through adapting (learning) processes may however lead to changes within the columns encoding for the other orientations over both short and long distances (cf. section Single Attributes). Each global cortical map (orientation, ocular dominance, direction of movement etc…) can be experimentally detected individually. For convenience, they are generally considered as if they overlap (Figure 5C). But, in fact, they are both anatomically and functionally tightly inter-linked in all layers of the visual cortex, which favors interactions (this underlies the main hypothesis advanced here).
- Patients with infantile strabismic
  *Of importance here, cortical maps are still present even in cases of infantile strabismus*. The retinotopic, orientation, spatial frequency and ocular dominance maps have been described in experimental animals (e.g., Löwel et al., 1998; Bosking et al., 2000; Engelmann et al., 2002; Schmidt et al., 2004; Schmidt and Löwel, 2006a,b, 2008). They have also been observed in humans with infantile strabismus (e.g., Barnes et al., 2001; Goodyear et al., 2002; Clavagnier et al., 2015). This is not a surprise since most of them (except the direction of movement and the high spatial frequency maps) are genetically programmed and are thus present even without any visual experience (Milleret et al., 1988; Crair et al., 1998; Crowley and Katz, 1999; Li et al., 2006, 2008; Mitchell et al., 2009; Tani et al., 2012; Smith et al., 2015b). Functional specificity of feed-forward connections as well as long-range intrinsic and interhemispheric ones is also still present within these maps. Thus, for example, columns activated by the same orientation remain preferentially inter-connected both within one given hemisphere and between the hemispheres (e.g., Schmidt et al., 1997). Correlating activities are also ensured through such connections between these columns (Roelfsema et al., 1994). But all of the cortical maps display abnormalities (whether observed in animals or humans) because their development depends on post-natal visual experience which has been incoherent and discordant because of the misalignment of the eyes. Thus, for example, some columns within the maps are poorly activated, in particular when activated by the amblyopic eye (Goodyear et al., 2002; Schmidt et al., 2004). In correlation with this, some columns within the cortical maps are smaller than normal, such as those for ocular dominance, in particular when activated through the amblyopic eye (Löwel, 1994; Goodyear et al., 2002; Crawford and Harwerth, 2004). Because of interocular suppression, the excitation/inhibition balance within these maps is also disturbed (Sengpiel et al., 1994, 2006; see also Scholl et al., 2013). Some columns are discordantly paired through cortico-cortical connections. Thus, for example, in infantile strabismus, intra-cortical horizontal connections extend primarily between neurons activated by only one eye (instead of binocularly driven neurons in normal conditions) which indicates that fibers between coactive neurons (from one eye) are abnormally selectively stabilized (Schmidt et al., 1997; Schmidt and Löwel, 2008). Correlated activities between columns activated by the same attribute within each map are still present but they are also reduced, in particular those between different ocular dominance domains (i.e., those activated by the left eye and the right eye respectively) since binocularity is reduced (Roelfsema et al., 1994). But, of great importance here, all this is malleable, even in the adult (discussed below).

Functional organization of visual cortical networks underlying integrated perception of various attributes of the visual scene

- Subjects with normal vision
  Considering the specific organization of both intra- and inter-hemispheric V_1_ neuronal networks implicated in the perception of the respective visual attributes (elaborated above), a key question then is whether and how the *principle of convergence* would apply there when *several* visual attributes are present, which is most often the case in natural environments. In fact, the *principle of convergence* applies in this situation despite its complexity, providing however the various inputs are coherent. This is indeed possible because: (i) each single neuron in V_1_ can be specifically activated by several visual attributes (cf. section Convergence at the Level of Single Neurons). Thus, each one is also included in multiple cortical columns and several global cortical maps for the respective attributes; (ii) all of those neurons which are simultaneously activated by the exact same visual attributes, i.e., which display exactly the same functional properties, are included within the same columns as well as within the same parts of the global cortical maps; (iii) the assemblies of neurons sharing the same functional properties are always preferentially interconnected whether considering one single or several visual attributes. Thus, for example, the *principle of convergence* will be adhered to by all of the neurons in V_1_ whose receptive fields overlap at the center of the visual field and which are activated by a thin vertical bar moving slowly rightward while the right eye is visually stimulated. They will indeed all be located in the foveal representation of the retinotopic map of V_1_. They will also be included in 3 sets of columns located in this same cortical region activated respectively by the vertical orientation (in the global orientation map), movements at 5 deg/s to the right (in the global map of direction of movement) and the right eye (in the global ocular dominance map). The same applies for all neurons whose receptive fields overlap within the right hemifield at 0° elevation and 30–40° lateral of the vertical meridian, and which are activated by a horizontally oriented border moving at 100 deg/s upwards, detected through the left eye only, etc. The number of possible combinations is enormous; the *principle of convergence* may thus apply in V_1_ to all sorts of neuronal networks activated by more or less numerous visual attributes, and implicated in the perception of more or less extended portions of the visual field. Of particular interest here, the *principle of convergence* thus also applies to extended neuronal networks in V_1_ while they are activated by various visual attributes present in large parts of the visual scene. Again, the preferred relations between the different columns activated by the same visual attribute within the cortical maps are implicated in this. But, additionally, both the long-range intra-cortical and inter-hemispheric connections will also contribute, allowing relations between extended and remote portions of V_1_. Thus, for example, interactions between regions V_1_ encoding portions of space separated by several degrees become possible (Gilbert, 1983 for review). This is thought to be crucial for elaborating a global perception of the visual scene taking into account both the elements “of interest” in the visual scene but also the context (see below).
  The *principle of convergence* does not apply here solely because neurons activated by the same visual attributes are preferentially inter-connected anatomically. *Synchronization* of oscillatory neuronal activity likely also plays a major role in this (cf. Milner, 1974; Von der Malsburg, 1981 for the theory). Wolf Singer and his collaborators were the first to support this view experimentally from their analyses of responses of single neurons in primary visual areas A17 and A18 of cats to single attributes. They indeed recorded synchronization of neuronal activity during the visual stimulation, mainly in the β and γ frequency ranges, i.e., 20–100 Hz (e.g., Gray et al., 1989; Engel et al., 1991; Fries et al., 2002; see also Singer and Gray, 1995; Singer, 1999, 2013; Engel et al., 2001; Fries, 2005; for reviews). These authors and many others further demonstrated that such oscillatory synchrony also applies to extended neuronal networks in visual cortex in general (i.e., in V_1_ and beyond) when activated by single visual attributes, in higher mammals including humans (Singer, 1999, 2013; Engel et al., 2001; Fries, 2005; for reviews). Very importantly here, in agreement with Milner and Von der Malsburg' theory (see above), these synchronizations are presently considered by Singer, his collaborators and many others to solve the “binding problem.” That is, they assemble all of the attributes of a visual scene into a single coherent representation for visual perception. These synchronizations are even considered as being able to take into account cognitive factors such as the context, attentional state, etc. (e.g., Singer, 1999, 2013; Fries, 2005; for reviews). Synchronization of different zones has also been recently demonstrated to predict perceptual content (Hipp et al., 2011) and, when abnormal, to be involved in brain pathologies such as schizophrenia and autism (Uhlhaas and Singer, 2006; Uhlhaas et al., 2009a,b, 2011). However, the role of synchronization during the elaboration of visual perception should be confirmed or at least it should not be presented as universal in the present context. This is because this view has been challenged by many other authors. Thus, for example, Thiele and Stoner (2003) found that perceptual binding of two moving patterns had no effect on synchronization of the neurons responding to the two patterns. In the primary visual cortex, Dong et al. (2008) found that whether two neurons were responding to contours of the same shape or different shapes had no effect on neural synchrony. Revonsuo and Newman (1999) reported similar negative findings. Without going into details, a number of highly recognized researchers such as Shadlen and Movshon (1999) and Merker (2013) have also raised concerns.
- Patients with infantile strabismus
  Considering the anatomical and functional organization of V_1_ in general, the *principle of convergence* may evidently also apply to visual neural networks of infantile strabismic patients underlying integrated perception of various attributes of the visual scene. However, not surprisingly, alterations in intracortical wiring and neural activity (e.g., Löwel and Singer, 1992; Schmidt and Löwel, 2008; see above for details) lead to reduced convergence and reduced neuronal synchrony in visual cortex relative to normal (Roelfsema et al., 1994). Recent data from the Hess team support this: they showed that interactions between cells in disparate brain regions are reduced when driven by the amblyopic eye of infantile strabismic subjects, from the dorsal lateral geniculate nucleus of the thalamus to higher visual areas, via V_1_ area (Li et al., 2011). They also demonstrated that amblyopia (in infantile strabismic patients) is associated to temporal synchrony deficits (Huang et al., 2012). This would then lead to altered visual perception, globally.
  - These data indicate that the *principle of convergence* we have introduced and tested here *applies to neuronal networks in V*_1_, whatever their size. Similar to single neurons, these networks are indeed also powerful *integrators* that are activated simultaneously by various attributes of the visual scene, providing, however, they work in a coherent way.
  - This holds true both in normally viewing and strabismic people, although the *principle of convergence* is applied more comprehensively in normal vision.

These conclusions are the first two inescapable conditions to support our hypothesis that present treatments of infantile strabismus may be improved. Area V_1_ indeed receives most (~90%) of the retinal inputs in higher mammals. And convergence needs to exit in it although it is mainly implicated in the decomposition of the visual scene in its basic features during the elaboration of visual perception (i.e., the so-called “segmentation” process).

## Convergence in superior visual cortical areas

**Figure 6:**
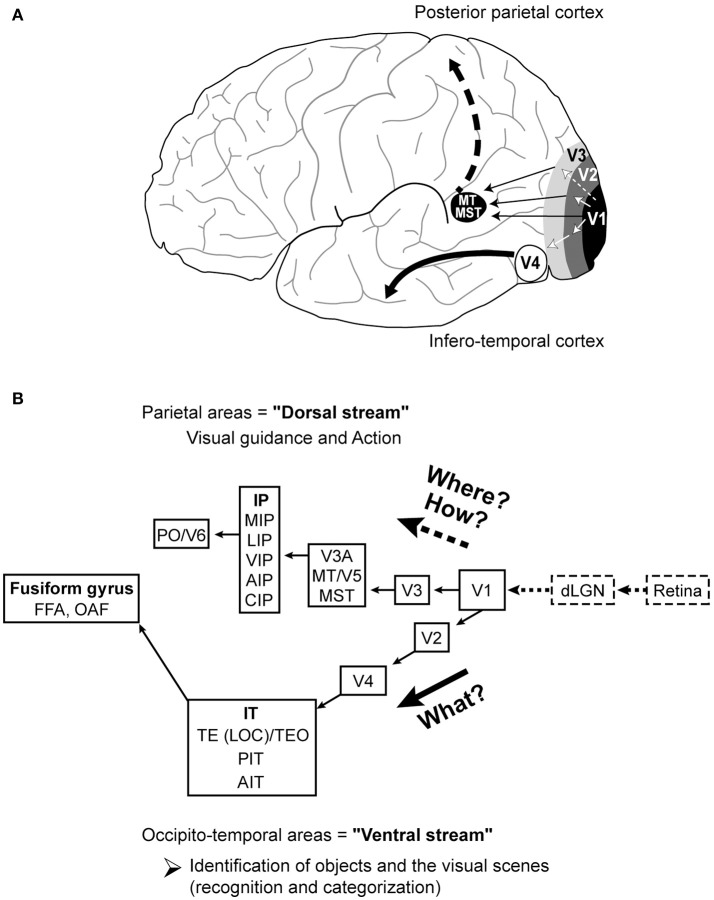
Dorsal and ventral visual streams. **(A)** Brain localization in humans. Starting with V_1_, the dorsal stream goes to posterior parietal cortex while the ventral stream goes to infero-temporal cortex. **(B)** The various areas included in the respective streams. *Dorsal stream*: V_3_; V_3A_; MT/V_5_, Middle Temporal area; MST, Medial Superior Temporal area; IP, intra-parietal area; MIP, medial intra-parietal area; LIP, lateral intra-parietal area; VIP, ventral intra-parietal area; AIP, anterior intra-parietal area; CIP, caudal part of the intra-parietal area; PO/V_6_, parieto-occipital area; *Ventral stream*: IT, infero-temporal cortex; TE (= LOC)/TEO, Inferior Temporal Areas; PIT, posterior infero-temporal cortex; AIT, Anterior infero-temporal cortex; FFA, fusiform face area, OFA, occipital face area. Note that both streams interact with one another (not represented here, but see Table 5 and text for details). Reproduced from Figure 2.4 in C. Milleret (2017), with permission from *Elsevier Masson* and copyrights.

Here we focus on V_1_ because it is activated by most of the retinal inputs (traveling through the geniculate pathway) in higher mammals and its anatomo-functional characteristics are rather well documented. But globally, there are more than 30 additional “superior” visual cortical areas in the so-called “visual cortex.” In the present context, this raises again the questions of whether and how the *principle of convergence* may also be applicable to each of these cortical areas and to each neural network in which they are included. Among other arguments to justify these questions, one may evoke the fact that similar to V_1_, each “superior” visual area includes neurons that are organized retinotopically, into “columns” and into “functional maps”. But they are each implicated differently in the elaboration of visual perception (see Table 2 for details). Also each may belong to different neural networks (Figure 6). They are all activated (directly or not) via V_1_ through “feed-forward” connections but each receives afferents from different other visual areas (Figure 6B). In most cases, each “superior” visual area also sends projections back through what are called “feed-back” connections but again these may project to different lower visual cortical areas, including V_1_ (Felleman and Van Essen, 1991). Beyond their specific cortico-cortical connections, some of these areas are also reciprocally interconnected with sub-cortical thalamic regions. As above, the amount of convergence will be compared successively in normally viewing subjects and those with infantile strabismus.

**Table 2:** Functional characteristics of *most* areas in the dorsal and the ventral streams of visual cortex in primates (including humans) illustrating the convergence of the various attributes.

	**Visual attributes**								**Main functions**
**AREA**	**RET**	**OR**	**SF**	**MV DIR**	**OD Δ d**	**3D**	**Color**	**Contrast**	
**V1**	+++	+++	+++	+++	+++	−	ε	+++	- Mainly implicated in the decomposition of the visual scene in its basic features during the elaboration of visual perception (i.e., the so-called “segmentation” process). - Neural basis for perception of local color contrast and color constancy (but not color by itself) by comparing color signals across visual space.
**Dorsal stream: “Where” system (Spatial perception**—**Localization) and “How” system (Non-conscious visually guided motor action)** Three pathways emerging from the dorsal stream additionally support both conscious and non-conscious visuo-spatial processing, including spatial working memory, visually guided action and navigation, respectively (not detailed here). Receiving mostly **M inputs**, but also P and K ones									
**V3**	+			++				+++	**Motion- selective area**—Quite high contrast sensitivity
**V3A**	+			+++	++	++		+++	**Motion- selective area**—**Projections to V3**—Quite high contrast sensitivity—Encodes Δd —Includes gaze-dependent neurons
**MT/V5**	+	+	+ LSF and HSP	+++	++	++	+	+++	**Detecting motion, direction, speed and speed gradient of motion**—**Plays a prominent role in the high-level perceptual analysis of gesture, namely the construction of its visual representation**—Contributes to the extraction of 3D shapes from 2D motion—Modulated by eye gaze but not by eye position—Activated during optokinetic stimulation. It is thus **fundamental for successful locomotion and processing of heading direction**. Also implicated in visually guided hand movements and the processing of heading direction—All this may occur unconsciously
**MST (V5a)**	+	+		+++		++			**Mainly involved in the analysis of the movements of the objects in external space**—**Also implicated in self-motion**—**Very implicated in the perception of the optic flow**—**Implicated in pursuit eye movements and saccades** >> **Fundamental for successful locomotion and processing of heading direction**—Some neurons respond to motion of a large patterned field while others respond preferentially to small spot motion—Neurons respond to 3D orientation of rotating planes – They may be sensitive to direction of motion—Implicated in the processing of heading direction—Multisensory cortical region—Tuned for direction of self-motion for both visual and vestibular modalities. – They may also respond to tactile stimulation
**IP** Including VIP LIP CIP MIP AIP	+	+		+++	++	+++			**IP: Very implicated in the control of visually guided actions like reach-to-grasp movements**—**Extracts the 3D shape and position of objects from 2D retinal images**. ***Sub-regions:*** **VIP:** Selective for smooth pursuit eye movements; sensitive to optic flow; implicated in self-motion; sensitive to backward or forward self-motion (contraction and expansion stimuli, respectively) and movement in the fronto-parallel plane; responds to horizontal rotations; responses to heading stimuli encoded in head-centered coordinates; multimodal parietal area; tuned for direction of self-motion for both visual and vestibular modalities. **LIP**: Includes robust neuronal category representations; participates to (but poorly) to object discrimination; activated by shapes (but less than IT); part is sensitive to depth of structures only and the 2D shape of small objects; Also: remaps visual stimuli traces in conjunction with eye movements; decodes target distances and saccade amplitude; is modulated by eye position; is considered as a neural basis for the control of an oculomotor brain-machine interface; Modulated during attentive fixation. **CIP:** includes neurons that are selective for orientation in depth of surfaces and elongated objects. **MIP:** involved in goal-directed arm movements and visuo-motor coordination; closely tied to decision-related motor actions; sub served visuo-motor coordinate transformation. **AIP:** very involved in processing 3D shapes; sensitive to depth structures and the 2D shape of small objects.
**PO/V6** (+ V6A)	+			+++		+++		+	**Motion area. One of the earliest stages underlying motion coherence. Is engaged in the spatial encoding of extra-personal visual space, i.e., ego-motion (** = **engaged in the processing of visual cues for self-motion). Acts as real-motion detector (** = **involved in the “recognition” of movement in the visual field). Selective for heading on the basis of the optic flow. Involved in distinguishing object and self-motion. Participates to the localization of targets and in arm-reaching for such targets. Has eye position-related activity**. Neurons respond to unidirectional motion (with a strong preference for elements with coherent motion). Highly selective for coherent moving targets >> very sensitive to translational motion. May distinguish between different 3D flow fields being selective to translational ego-motion. Visual responses modulated by eye position in the orbit. Action quite uniform in the area. Some (but not all) RFs move with gaze according to eye displacements, remaining at the same retinotopic position (usual for visual neurons) >> Includes both eye position-sensitive neurons and neurons encoding real positions. V6A includes neurons related to the control of arm movements
**Ventral stream – “What system”** >>> **Perception of shapes and object recognition** Receiving mainly **P** and **K** inputs but also some M ones									
**V2** Including 5 afferent streams	+	+	+	+	++		Clue to hue	+	**Implicated in Figure-ground organization**. Emergence of proto-objects, the natural scene thus becoming effective. Activated by luminance-defined and texture-defined forms (+ their orientation). Activated by some aspects of color vision: clue to hue (through glob cells). Receives inputs from parietal areas which may potentially bring contribution of attention during active vision
**V4**	+ Poste-rior Part only	+	+ (LSF + HSP)	+	++		+++	+	**Shape perception: well activated by object contours features (angles and curves). Color vision. Also includes direction-preferring domains (forming columns and a map), tending to overlap OR and color-preferring domains**. Accurately represents isolated shapes in terms of their component contour features. Provides a percept of partial occlusion (when objects have accidental contours). Contributes to both single- and multiple-viewpoint shape discriminations. **Influences the oculomotor planning process during natural vision** through convergence of bottom-up and top-down processing streams. Saccade preparation also modulates the visual responses of neurons by the deployment of attention
**IT** TEO PIT TE (= LOC) AIT	+		+	+	+	+++	+	+	**Visual pattern perception and object recognition including faces (sometimes to only one or a few individuals), animals, man-made objects (with each encoded in a different region, with however a columnar organization in each case that is proto-organized, i.e., genetically programmed). Area where 3D structure is extracted from disparity**. Neurons signal global content of a hierarchical display before they signal its local contents (the forest is seen before the trees). Individual neurons are activated first by partial and simple aspects of the objects. Then IT responds to complex shapes, images of faces and hands whatever the angle of presentation and their size. Responses to compound objects are elaborated such a way that the whole is equal to the sum of the discrete parts. The medial axis of shapes in high-level object is however encoded specifically. Neurons may encode the real size of the objects. Image familiarization sharpens response dynamics of neurons in IT. Face-selective neurons form patches of various sizes distributed throughout IT. Face and object selectivity are organized spatially with a fine scale
									**Interactions in IT:** Context influences beyond the “classical” RF (Spillmann et al., 2015) —Texture discrimination in IT predicts texture perception (Zhivago and Arun, 2014) —SF components influences cell activity in IT—These shapes may be defined by relative motion or by texture differences—IT responds to disparity-defined 3D shapes—IT sub-serves 3D structure categorization—Integrates color perception in the context of behavior—Neurons respond even in case of partial occluded shapes—Linear addition of shape and color signals (McMahon and Olson, 2009) —Contrast is important for identifying face (Ohayon et al., 2012) **AIT:** Face-responsive neurons are most often tuned around an average, identity-ambiguous face—Individual faces elicit distinct responses patterns—Neurons engaged in object recognition can be highly sensitive to object retinal position—Allows fine shape distinctions—Spatial information is available for the representation of objects and scenes within a non-retinotopic frame of reference—Modulated by eye position. **TE (monkey):** Responds to both local and global complex visual objects features—Those neurons that respond to similar features are organized into columns—**Extracts 3D structure from disparity**—Encodes color (anterior temporal cortex) **LOC (human):** Responds more strongly to shapes than to edges, surfaces, random stimuli or holes, for both motion and stereo cues. Thus LOC processes shapes (not edges or surfaces) and some border aspects. **TEO and TE:** Integrate information about multiple contour elements (straight and curved edge fragments of the type represented in low-level areas) using both linear and nonlinear mechanisms
**Fusiform gyrus** FFA, OFA Strongly right lateralized				+			+++		**Mostly involved in face perception and recognition. But also involved in body and objects perception** (with each being treated in a separate portion of the fusiform area) —**Also involved in color perception FFA and OAF (human):** Independent populations of neurons which are specifically activated by faces, parts of faces, and head outlines.

*Abbreviations: visual cortical areas, same as in Figure 6; RET, OR, SF, MV, DIR, and OD, same as in Table 1. Δd, binocular disparity; 3D, depth perception (= stereopsis); LSF, low spatial frequency; HSF, high spatial frequency. As a general rule, receptive sizes increase beyond V_1_. During behavior, attention generally participates in the elaboration of visual perception. Empty columns indicate that data was not found. Sources cited here are not in the List of references. The interested reader is invited to search in the reference list. Dorsal stream: Ferrera et al. (1994), Greenlee (2000), Grefkes and Fink (2005), Orban et al. (2006), Orban (2008), Britten (2008), Wall and Smith (2008), Angelaki et al. (2011), Orban (2011), Kravitz et al. (2011), Angelucci and Rosa (2015), Skottun (2015). Ventral stream: Ferrera et al. (1994), Vogels and Orban (1996), Bartels and Zeki (2000), Sincich and Horton (2005), Zangenehpour and Chaudhuri (2005), Orban et al. (2006), Pasupathy (2006), Reddy and Kanwisher (2006), Connor et al. (2007), Gross (2008), Orban (2008), Conway (2009), Conway (2014), Federer et al. (2009), Sereno and Lehky (2011), Taylor and Downing (2011), Roe et al. (2012), Sato (2012), Kravitz et al. (2013), Collins and Olson (2014), Xiao (2014), Skottun (2015), Lehky and Tanaka (2016)*.

### Subjects with normal vision

#### Evaluation of convergence in each superior visual cortical area

Most of the “superior” visual areas are listed in Table 2. For each, we have indicated the effective visual attributes and the resulting main functions. Thus, for example, the intra-parietal area (IP) displays a retinotopic organization and includes neurons which are activated by the orientation, the velocity/direction of movement and the position disparity of the visual stimuli. Accordingly, it contributes to extract the 3D shape and position of objects from 2D retinal images. As another example, V_2_ may be activated by almost all the visual attributes. It is mainly implicated in figure-ground organization, i.e., the tendency of the visual system to simplify a scene into the main object that we are looking at (the figure) and everything else that forms the background (or ground). In fact, various (but in general not all) attributes converge onto each of these superior visual areas. But the more the area is distant from V_1_ (cf. Figure 6B), the more it contributes to the elaboration of visual perception in an increasingly complicated manner.

- The *principle of convergence* is respected within each of these superior visual cortical areas although fewer visual attributes converge on them compared to V_1_. This is coherent with the fact that each of these “superior” visual areas contribute specifically to the elaboration of visual perception (see also below).

#### Cortico-cortical feed-forward connections

The superior visual cortical areas may receive their inputs from V_1_ through two main pathways: either the *dorsal* or the *ventral* streams (Figure 6A; see Ungerleider and Haxby, 1994; Van Essen and Gallant, 1994; Shen et al., 1999 for reviews). Each includes different visual cortical areas which contribute to different aspects of the elaboration of visual perception (see just below for details). Whether and how the *principle of convergence* may still apply at the level of each of these streams again needs to be evaluated in the present context. Because of their complexity and limited space, only major properties and representative data are summarized below, with a few examples. More details including references are provided in Table 2.

*The dorsal stream*. extends from V_1_ to the posterior parietal lobe. It includes many visual areas such as V_3_, V_3A_, MT/V_5_, MST (V_5A_), IP (sub-divided at least in MIP, LIP, VIP, AIP, CIP) and PO/V_6_ which thus globally participate to more and more highly specialized and elaborated aspects of visual perception. Each area thus has specific functions which complement one another (cf. Figure 6B; see also Table 2 for details, including a key to these abbreviations). Thus, for example, the *Middle Temporal area (MT/V*_5_*)* is mostly specialized in analyzing motion (detection, direction and speed including gradients). It contributes to extracting 3D form information from 2D motion of an object. It plays a prominent role in the high-level perceptual analysis of gestures, namely the construction of its visual representation. In addition, it is modulated by gaze direction and is activated during optokinetic stimulation. Complementing this, the *Medial Superior Temporal area (MST)* is mainly involved in the analysis of the movements of objects in space. It is also highly implicated in the perception of self-motion and optic flow (i.e., an apparent motion of visual space in the opposite direction to the movement of the subject). MST is also implicated in regulating pursuit eye movements and saccades. Both areas are consequently fundamental for the perception of movement including direction of movement, processing heading direction, and thus are involved in locomotion but each clearly contributes in a different way.

Altogether, the dorsal stream responds to the question “Where?” with respect to objects in space and thus is vital for *spatial perception* and *spatial localization*. It also helps respond to the question “How?” by dealing with *visually guided action*, that is, the subject's orienting movements relative to images localized in space. As summarized in Table 2, to ensure all these functions, the dorsal stream is more activated by the M (magnocellular) pathway. This is in agreement with the fact that it is the “movement” here that is mostly concerned. But it is also activated through the P (parvocellular) and K (koniocellular) pathways which provide additionally information about other visual attributes, i.e., the location in space, orientation, spatial frequency, ocular dominance, binocular disparity, depth perception, color and contrast. Information about hand and head position also have to be added. Since each area included within this stream is activated differently by the different visual attributes (cf. section Evaluation of Convergence in Each Superior Visual Cortical Area), each is also differentially activated by the M, P, and K pathways.

- Thus, the *principle of convergence* of different attributes of the visual scene also applies to the dorsal stream of the visual system in higher mammals.

*The ventral stream*. runs from V_1_ to the infero-temporal lobe (Figure 6A). It includes various areas such as V_2_, V_4_, IT (sub-divided at least into TE (LOC)/TEO, PIT, and AIT regions) and the fusiform gyrus (including at least the FFA and the OAF areas) and thus is distinct from the dorsal stream. Similar to the latter, however, these areas globally participate to the elaboration of a more and more complex aspects of visual perception. Thus, for example, *V*_4_ is activated by objects' contour features such as curves and angles. It contributes to both single- and multiple-viewpoint based on shape discrimination. It is here that *true* color vision emerges, meaning for example that long and short wavelengths are now perceived respectively as “red” or “blue.” But it also includes neurons preferentially activated by one given direction of movement and influences oculomotor planning processes. *The Infero-Temporal area (IT)* is one of the highest visual cortical areas. It is implicated in identification of faces, animals, man-made objects, etc. IT is also the visual area where 3D structure is extracted from binocular disparity. Both V_4_ and IT belong to the “What?” system since they are clearly implicated in the perception of shapes and recognition of objects.

As summarized in Table 2, to ensure these functions, the ventral stream mainly receives P and K inputs (for the elaboration of perception of shapes and color). But it also receives M inputs to encode movements. Again, information about various attributes of the visual scene are needed in order that each area in this stream carries out its own function. Since they differ from one area to the other, this indicates again that each is differentially activated through the P, K, and M pathways.

- The *principle of convergence* is thus also respected in the ventral stream, even if the processes are more complex than in V_1_.

#### Cortico-cortical feed-back connections

Higher visual cortical areas such as V_2_, V_3_, V_4_, and MT/V_5_ areas project back to V_1_. In the hierarchy of the cortical areas, the feed-forward connections are broadly defined as those that terminate mainly in layer IV (see Figure 3). In contrast, the feed-back connections originate from neurons in superficial and/or deep layers and terminate mainly outside layer IV. The most superficial terminal fields exhibit a very precise “point-to-point” retinotopic connectivity while the deeper ones display a more diffuse organization (e.g., Batardiere et al., 1998; Markov et al., 2014). Some authors have even shown that the most superficial subset of feed-back connections terminate in a patchy fashion in layers II-III (Inter-blob zone), IV-B and V-VI of V_1_, and show modular and orientation specificity while the deepest ones are not only diffuse, but also unspecific and strictly terminating in layer IA (Angelucci et al., 2002; see also Angelucci and Bressloff, 2006 for review). Note that some exceptions to this rule have however been reported with some spatial overlap of feed-forward and feed-back terminals (Angelucci et al., 2002).

At least from our knowledge, the contributions of the feed-back connections during the global elaboration of visual perception are fundamental but complex and far from being completely understood. For example, Gilbert and Li wrote that such connections *likely* “carry rich and varied information about behavioral context, including attention, expectation, perceptual tasks, working memory and motor commands” (Gilbert and Li, 2013), but this remains to be clarified. On the basis of various studies however, such connections are at least recognized as contributing to *refine* and *modulate* neural activity in the lower structures. This has been demonstrated in V_1_ unit recordings, where feed-forward intra-cortical horizontal connections and feed-back connections from superior visual cortical areas play complementary roles during the elaboration of visual perception (e.g., Stettler et al., 2002; Schwabe et al., 2006; Piëch et al., 2013; Ramalingam et al., 2013; discussed further below).

Altogether, the feed-back connections likely contribute to perceptual grouping of the elements of a single object that are represented in a fragmented manner by neurons with limited receptive fields. This includes contour integration, distinguishing contextual interactions and perceptual segregation of overlapping objects, including perceptual discrimination accuracy. Their implication in perceptual grouping and contextual interactions at least evidently implies that such feed-back connections necessarily favor a coherent convergence of information within V_1_ area during the elaboration of visual perception, at least by some aspects.

- Thus, for coherence, even if this question has not been investigated specifically yet, our guess here is that the *principle of convergence* needs to be also respected by the feed-back connections.

#### Trans-thalamic cortical routes

The superior visual cortical areas are also reciprocally connected with various sub-cortical thalamic regions belonging to the extra-geniculate nuclei complex (e.g., thalamo-cortical: Berman and Wurtz, 2011; Gamberini et al., 2016; cortico-thalamic: Ungerleider et al., 1984; Van Essen, 2005). Such trans-thalamic cortical routes also contribute in some ways to the elaboration of visual perception. Thus whether and how the *principle of convergence* may still apply at their level needs to be examined as well. We cannot analyze each of these trans-thalamic cortical routes here. Anyway, most of them are not totally characterized. Thus, to approach this question, we have rather decided to focus on the representative route formed by MT/V_5_ and its reciprocal connections with the pulvinar nucleus located within the thalamus (e.g., Ungerleider et al., 1984; Shipp, 2001; Berman and Wurtz, 2008; for review, see Casanova, 2004). Both of these structures are strongly implicated in higher-order motion integration (MT/V_5_: cf. section Cortico-Cortical Feed-Forward Connections and Table 2 for details; pulvinar nucleus: Villeneuve et al., 2005; see also Merabet et al., 1998; Casanova et al., 2001; Dumbrava et al., 2001).

As shown above, MT/V_5_ already benefits from considerable convergence through the dorsal stream although it is mostly activated via the M pathway (cf. section Cortico-Cortical Feed-Forward Connections and Table 2 for details). The same holds true for the pulvinar nucleus which is even considered as a “connectional hub” and a major “relay” between the thalamus and the cortex (Bridge et al., 2016). The convergence of abundant visual information is more especially ensured in the pulvinar nucleus by its reciprocal connections with many superior visual cortical areas including V_2_, V_3_, V_3A_, V_4_, MST, and FST, in addition to MT/V_5_, belonging to both the dorsal and the ventral streams (e.g., Grieve et al., 2000; Van Essen, 2005; Arcaro et al., 2015; for review, see for example Bridge et al., 2016). Thus the pulvinar nucleus likely receives information from all three visual pathways (M, P, and K) through these reciprocal cortico-thalamic connections and integrates all of the visual attributes, even if it has been more implicated in motion perception. Note that, in complement, the pulvinar nucleus receives retinal afferents through the extra-geniculate pathway, originating at the level of the optic tracts. These afferents project from the retina either *directly* or *indirectly* through the Superior Colliculus (located in the midbrain, also partially implicated in vision) to different parts of the inferior pulvinar nucleus, with the indirect ones being likely dominant (e.g., Berman and Wurtz, 2011; Bridge et al., 2016; Kwan et al., 2018). In agreement with the visual information being exchanged between the pulvinar nucleus and the visual cortex (see above), these retinal fibers apparently comprise axons of all three major ganglion cell classes, i.e., at the origin of the M, P, and K pathways (e.g., Cowey et al., 1994).

Altogether, these data indicate that the *principle of convergence* may also be respected within the trans-thalamic cortical routes. On the basis of the above described example, this convergence is even likely very extensive suggesting an implication of these routes in many (but not yet completely identified) situati***ons*.**

### Infantile strabismic patients

Consistent with functional changes in V_1_, superior visual cortical areas of both the dorsal and ventral streams are also affected after infantile strabismus (e.g., Thompson et al., 2012 who conducted their study in humans). Thus, for example, it has been established that global and local perception of forms, including faces, is altered; both global and the local motion perception is altered as well (e.g., Birch, 2013; Hamm et al., 2014; Levi et al., 2015 for reviews). But also, similar to V_1_, their general anatomo-functional organization remains unperturbed since it mainly results from innate and genetic factors. Note that although little is known about changes in feed-back connections and trans-thalamic cortical routes in infantile strabismus, our guess is that the same applies to them. One may underline however that Thompson et al. recently reported unexpected but very interesting findings concerning the latter connections, more specifically the ones between MT/V_5_ and the pulvinar nucleus (cf. section Trans-thalamic Cortical Routes). In the light of previous work indicating a functional MT/V_5_ deficit in case of amblyopia, they first conducted a psychophysical experiment investigating the ability of amblyopic eyes to perceive coherent and incoherent plaid stimuli (Thompson et al., 2008). To their surprise, however, they found that amblyopic eyes mediated largely normal perception of both coherent and incoherent plaids. They thus proposed two possible (non-exclusive) explanations for this finding. The first was that the integration of local motion signals in MT/V_5_ is abnormally susceptible to noise in amblyopia and it is the noise in the stimuli rather than the motion integration deficit *per se* that causes the impaired performances (Mansouri and Hess, 2006). *The second explanation was that an alternative network of neural areas is recruited to support normal plaid perception by the amblyopic eye*. Candidate areas included those that have been shown to process with selectivity for coherent plaid motion, i.e., V_1_ (Guo et al., 2004), V_3_ (Gegenfurtner et al., 1997; Wenderoth et al., 1999) and the pulvinar nucleus (Merabet et al., 1998; Villeneuve et al., 2005). To resolve this, Thompson et al., used functional magnetic resonance imaging (fMRI) to compare responses in the visual cortex and thalamus to incoherent and coherent motion of plaid stimuli while they were perceived normally by the amblyopic eye or the non-amblyopic eye (Thompson et al., 2012). Without entering into the details, their results have indicated that while the perception of the plaid stimuli was constant for both amblyopic and non-amblyopic viewing conditions, the network of neural areas that supported this perception was different. Both cortical responses in MT/V_5_ and sub-cortical responses in the pulvinar nucleus were correlatively different. To interpret these data, the authors propose that the visual system of strabismic subjects with amblyopia is able to compensate the MT/V_5_ deficit by recruiting an alternate network to perceive correctly higher-order motion. The pulvinar nucleus was considered as a very good candidate for that although it might be confirmed in this function.

The *principle of convergence* is maintained in infantile strabismus, even if the neural bases for perception of respective attributes of the visual scene may be altered. This is reinforced by additional data (in particular from psychophysical measures) reported below.

As a general conclusion:

- The *principle of convergence* may be extended to all visual cortical areas and the neural networks in which they are included both in normally sighted subjects and those with infantile strabismus. In each case, the elaboration of visual perception is achieved through a coherent link of their activity even some abnormalities exist after strabismus.
- To support our hypothesis, such convergence of information about the different visual attributes both in each superior visual area and within the neural networks in which they are included is also one inescapable condition to consider that the present treatments of infantile strabismus may be possibly improved compared to presently.

## Principle of interactions and of inter-dependency of all the attributes of the visual scene

As developed in section Principle of Convergence in Visual Cortex, the *principle of convergence* is applicable at any level (neurons, columns, cortical maps and neural networks) in the whole visual cortex both in normally viewing and infantile strabismic subjects, even though the latter have some weaknesses. Indeed, strabismic subjects are still able to quite coherently perceive visual scenes in spite of their visual system abnormalities.

Thus, *interactions* are facilitated between the various attributes for visual perception whether vision is normal or not. All of the attributes of the visual scene are also *inter-dependent*. This is fortunate from the perspective we are developing here since *interaction* and *inter-dependency* of the various attributes of the visual scene, at any level that they may occur, are also major keys here for considering that rehabilitation of full visual function may be possible after infantile strabismus. This is illustrated below in higher mammals including humans, both during post-natal development and in the adult, through functional and anatomical data obtained by exploring V_1_ and superior visual cortical areas as well as through psychophysical data. Data from mathematical modeling will also be evoked. As usual, we start with subjects with normal view and then discuss subjects with infantile strabismus.

### Subjects with normal view

#### Interactions and inter-dependency during post-natal development

First, it is important to emphasize here that the *principle of convergence* applies to the post-natal period of development. For half a century, we have been known that each neuron in V_1_ responds to each visual attribute from birth or soon after birth from the day the eyes open (e.g., Hubel and Wiesel, 1963b; Frégnac and Imbert, 1978; Milleret et al., 1988, 1994). A cortical map is also initially present for each attribute of the visual scene (except apparently for direction of movement and high spatial frequencies; see above). The visual responses are initially quite poor and sluggish but they get increasingly stronger and are refined with post-natal visual experience (e.g., Chapman et al., 1996; Crair et al., 1998; Crowley and Katz, 1999; Tani et al., 2012). In parallel, the cortical feed-forward and feed-back networks also develop and refine both anatomically and functionally (e.g., Movshon and Van Sluyters, 1981; Wiesel, 1982; Garey and de Courten, 1983; Innocenti, 1986; Wang et al., 2016). The same holds true for the trans-thalamic cortical routes (Bridge et al., 2016). Interactions and inter-dependency between the different visual attributes thus exist from the earliest stages after birth.

On the basis of the anatomo-functional organization of V_1_ reviewed above, not surprisingly, a key initial characteristic of V_1_ neurons is their potential to interact and their inter-dependency over short and long distances in cortex even when considering *only a single attribute* of the visual scene. This was investigated for example by Schuett et al. (2001) who studied plasticity of orientation preference maps in A17 of kittens (i.e., V_1_ homolog). Pairing a brief visual stimulus of a given orientation (OR_1_) with electrical stimulation of the cortex in a region underlying another orientation (OR_2_), they found that their relative timing determines the direction of plasticity: the recorded neuron changed its orientation preference (OR_1_) toward the orientation of the point that was stimulated (OR_2_) if the cortex was activated first visually and then electrically; by contrast, the cortical response to the paired orientations was diminished if the electrical activation came first. This allowed demonstrating interactions and inter-dependency between columns underlying different orientations within the global orientation map. In addition, they showed that pinwheel centers (singularities in the orientation cortical map; Figure 5B) are less affected by the pairing than the surrounding orientation columns which allowed to conclude that plasticity is however not uniformly distributed within the cortex during the critical period.

Furthermore, the development of the cortical maps corresponding to the *different visual attributes* may be inter-dependent. For example, Tani et al. (2012) investigated kitten's primary visual cortex with optical imaging of intrinsic signals and showed that the maturation of the orientation, spatial frequency and retinotopic maps are tightly linked. Among other findings, they indeed demonstrated that orientation maps for lower spatial frequencies appear first in the whole retinotopic map (where the whole visual field is represented) while the orientation maps for high spatial frequencies appear only later in correlation with the maturation of the part of the retinotopic map where the central vision is treated (Tusa et al., 1981). Inter-dependency was also shown by Wang et al. (2010) who reported that orientation preference in V_1_ (of mice) matches binocularly only by the end of the critical period. Through an electrophysiological approach, they indeed observed that the preferred orientation of individual cortical cells is mismatched between the two eyes soon after birth but that the binocular similarity of orientation preference improves and reaches adult levels during the critical period, i.e., with post-natal visual experience (see also Espinosa and Stryker, 2012 for review). This implicitly indicates that orientation preference and ocular dominance are inter-dependent during development.

- Thus, as early as the neural bases of responses to a given attribute develop, this has an impact on the development of the neural bases of other visual attributes. However, the time course of their respective critical periods has to overlap (see above).

#### Interactions and inter-dependency in the adult

##### Single attributes

In V_1_, interactions concerning a single attribute occur first at the level of single neuron, because of the organization of its receptive field. As mentioned in section Convergence at the Level of Single Neurons, each receptive field includes at least a “classical” part as defined by Hubel and Wiesel (1962, 1965) evoking spike activity and a larger surrounding “silent” zone sensitive to visual stimulation but without generating any spikes (Bringuier et al., 1999). More recently, three distinct regions have in fact been distinguished (Angelucci et al., 2002; Angelucci and Bressloff, 2006 for review): (i) The “classical” receptive field (or receptive field center); (ii) The “spatial summation” receptive field which also generates spikes during visual stimulation but whose size is contrast dependent when mapped with an optimal size stimulus: smaller when highly contrasted, but larger with low contrast; (iii) The “surrounding” (silent) receptive field which may be inhibitory or excitatory. The latter study also showed that feed-forward retino-geniculo-cortical pathways produce both the “classical” receptive field and the “high-contrast spatial summation” receptive field while intra-cortical feed-forward connections and feed-back connections from higher visual areas produce the “low-contrast spatial summation” receptive field and the “surrounding” (silent) receptive field respectively. These receptive field types in V_1_ are evidence of modulation by contextual stimuli (belonging however to the same attribute family) lying near or far outside the “surrounding” (silent) receptive field. This has been demonstrated for orientations since responses to discrete stimulation within the “classical” receptive field (via light bars or Gabor patches) are often facilitated by iso-oriented stimuli presented in the “high-contrast spatial summation” receptive field while they are usually suppressed when presented in the “surrounding” (silent) receptive field (e.g., Blakemore and Tobin, 1972; Nelson and Frost, 1978; Gilbert and Wiesel, 1990; DeAngelis et al., 1994; Mizobe et al., 2001; Chisum et al., 2003). A specific case of short-range modulation is “co-linear facilitation,” a phenomenon which is thought to underlie perceptual grouping of contour elements (Kapadia et al., 1995; Hess and Field, 1999). The fact that the most superficial feed-back projections terminate in layers II-III of V_1_ in a patchy fashion, showing modular and orientation specificity (see above) is consistent with this proposed role in orientation-specific center-surround interactions. Such latter interactions have also been demonstrated for contrast detection (Levitt and Lund, 1997; Polat et al., 1998; Mizobe et al., 2001). Note that center/surround organization of each receptive field also exists in superior visual areas, for example when encoding of direction of movement and velocity of movement of visual stimuli in MT area is under control sRF (Allman et al., 1985).

Interactions and inter-dependency concerning each single attribute evidently also occur at the level of visual cortical maps. A first example is adaptation-induced plasticity of orientation observed during electrophysiological recordings and intrinsic signal imaging of cortical maps in V_1_ of adult cats. Following prolonged exposure to a single oriented stimulus, changes of the neuronal responses to orientation occur and the direction of the shifts toward (attractive) or away (repulsive) from the adapter in the orientation global map depends on adaptation duration. This capacity for adaptive changes of the neurons is again not uniformly distributed in visual cortex, i.e., being the highest in iso-orientation domains and the lowest at and near pinwheels. Synchronous firing of orientation selective neurons is also modified during adaptation-induced plasticity (e.g., Dragoi et al., 2000, 2001; Schummers et al., 2005; Ghisovan et al., 2008, 2009; Nemri et al., 2009; Cattan et al., 2014). Experimentally induced synchrony also induces major changes in the orientation maps in adult cats (Godde et al., 2002). Such adaptive processes involve interactions over short and long distances through cortico-cortical connections (Yousef et al., 2001). In summary; adaptation-induced plasticity in the adult visual cortex is a dynamic time-dependent process that involves network interactions and that can lead to response depression and enhancement. A second example is the implication of the feed-back connections from superior visual areas in shaping visual cortical maps in V_1_, in particular the orientation and direction of movement maps. Suppressing feed-back signals from the posterior parietal cortex in the cat even abolishes the global layout of the direction of movement maps (e.g., Galuske et al., 2002; Tong et al., 2011). With fMRI in humans, Williams et al. (2008) also demonstrated that the foveal zone of V_1_ is sensitive to information about objects presented in the periphery because of feed-back connections from superior visual cortical areas (see also Yu and Shim, 2016).

*These data show how interactions in visual cortex allow local signals concerning one attribute to be integrated across visual space to generate global percepts as well as how they contribute to perceptual figure-ground segmentation of visual scenes and contextual modulation (e.g., Gilbert et al.*, *1996**; Seriès et al.*, *2003**)*.

##### Interactions and inter-dependency between various visual attributes

Interactions and inter-dependency between pairs of attributes. There is also evidence that interactions and inter-dependency exist between each pair of visual attributes (see Table 3 and IT section of Table 1). For example, these have been described for spatial frequency and contrast (Enroth-Cugell and Robson, 1966; Campbell and Maffei, 1981), spatial frequency and motion (e.g., Bisti et al., 1985), form (= orientation + spatial frequency) and color perception (e.g., Moutoussis, 2015), ocular dominance and orientation (Crair et al., 1997; Nakagama et al., 2006) as well as for spatial location (retinotopy) and velocity of movement (e.g., Orban et al., 1978).

**Table 3:** Examples of interactions and inter-dependency of pairs of visual attributes in adults with normal vision.

**Interactions**	**Main results**	**References**
RF size/Contrast	The static RF features vary with contrast and contrast adaptation in V_1_ of macaque.	(Durand et al., 2012) (P)
OR/Contrast	See section Single Attributes Neurons in primate V_1_ and V_2_ responding selectively to orientated luminance contours and neurons signaling non-luminance defined contours are distributed invariably in all the OR columns	(Angelucci and Bressloff, 2006) (M) (An et al., 2014) (OI)
SF/Contrast	The sensibility to contrast (1/C) varies with SF with an optimal at a certain SF	(Enroth-Cugell and Robson, 1966) (P); (Campbell and Maffei, 1981) (PSY)
OR/RET	Both OR and RET maps are inter-dependent: Long-range horizontal connections in V_1_ preferentially link neurons with co-oriented and co-aligned RFs Distortions of visuotopic map in V_1_ match orientation singularities (PWs)	(Bosking et al., 1997) (OI + Anat) (Das and Gilbert, 1997) (OI) (Yu et al., 2005) (OI) (Martin et al., 2014) (OI + Anat)
	Alternative view: both OR and RET maps in V_1_ are independent	(Buzás et al., 2003) (OI) (Paik and Ringach, 2012) (M)
SF/OR	Neurons in V_1_ with the same optimal SF are aligned orthogonal to the OR columns SF and OR maps in V_1_ are organized orthogonal to each other Optimal SF tends to remain constant whenever the preferred OR rotates progressively from cell to cell in V_1_ A local maximum and/or a minimum in the SF map exists around PW centers (= pinwheel-dipoles)	(Berardi et al., 1982) (P) (Nauhaus et al., 2012) (2P) (Ribot et al., 2013) (OI + M) (Romagnoni et al., 2015) (M) (Ribot et al., 2016) (OI + M)
OR/Movement (TF)	An apparent speed (perceptive illusion depending on the context) is OR dependent Shape and motion interact at perceptual and attentional levels during processing of structure from motion stimuli Brain activity is higher during perceptual form/motion integration (binding) than during segmentation of each visual attribute Perceptual alterations between unbound moving contours and bound shape motion engage a ventral/dorsal interplay Local form-motion interactions influence global form perception β but not γ band oscillations index visual form-motion integration Local (OR) and global (MV) information are already linked in V_1_	(Seriès et al., 2002) (PSY) (Georges et al., 2002) (PSY) (Miskiewicz et al., 2008) (PSY) (Aissani et al., 2011) (MEG-PSY) (Caclin et al., 2012) (PSY) (McCarthy et al., 2012) (PSY) (Aissani et al., 2014) (PSY) (Gérard-Mercier et al., 2016) (P)
Natural scene/MV	Optimal speed estimation in natural image movies predicts human performance	(Burge and Geisler, 2015) (PSY)
Form/Color	Chromatic information supports form perception	(Moutoussis, 2015) –Review (PSY)
Motion/Color	Sensitivity to chromatic motion is strongly marked by either luminance or chromatic contrast	(Webster and Mollon, 1997) (PSY)
OD/OR	OD peaks at PWs center singularities of the OR map in V_1_	(Crair et al., 1997) (OI) (Nakagama et al., 2006) (OI + M)
OR/ON-OFF	OR maps have a retinal origin (i.e., ON- and OFF-center retinal ganglion cells) The topography of ON and OFF inputs in V_1_ enables an invariant columnar organization	(Paik and Ringach, 2011) (M) (Lee et al., 2016) (OI)
Motion/depth	At moderate pursuit velocities, depth thresholds are limited by motion signals	(Holmin and Nawrot, 2015) (PSY)
SF/MV	SF in V_1_ depends on the motion velocity (MV) of the visual stimulus	(Bisti et al., 1985) (P)
Motion/Color	Color and motion are linked during visual perception But whether the binding between both occurs as early as V_1_ is still under debate	(Blaser et al., 2005) (PSY) (Linares and López-Moliner, 2006) (PSY) (Zhang et al., 2016) (PSY)
RET/Motion	Velocities encoded in visual cortex increase with eccentricity within the visual field (i.e., larger in periphery than centrally)	(Orban et al., 1978) (P)

*RET, OR, SF, MV, DIR and OD, same as in Table 1. RF, receptive field; PW, pinwheel; TF, temporal frequency. P, physiology; OI, optical imaging; 2P, two-photon microscopic imagery; MEG, magneto-encephalography; ANAT, anatomical study; PSY, psychophysics; M, models*.

Interactions and inter-dependency between triplets of visual attributes. Ophthalmologists, orthoptists and optometrists presently concentrate their efforts on the three following visual attributes and their inter-dependency in patients with infantile strabismus: visual acuity of each eye, binocularity and 3D perception, with the binocularity being associated here to the alignment of the eyes through surgery. But interactions and inter-dependency between other triplets of visual attributes have also been described. Thus, for example, as illustrated in Table 4, the trios “orientation, ocular dominance and spatial frequency” (Hübener et al., 1997), “orientation, direction of movement and ocular dominance” (Kim et al., 1999; Buzás et al., 2001) “direction, spatial frequency and contrast” (Lalanne and Lorenceau, 2006) as well as “spatial frequency, temporal frequency (i.e., motion) and color” (Shoham et al., 1997) are also tightly inter-linked. In fact, because of the extensive convergence of visual information within the whole visual cortex, all possible combinations likely lead to interactions.

**Table 4:** Illustrating interactions (or frames of interactions) occurring between 3 and 4 visual attributes in normally viewing adult: RET, OR, PW, SF, MV, DIR and OD; P, OI, 2P, ANAT, PSY, M, same abbreviations as in the other Tables.

**Interactions**	**Main results**	**References**
OR/OD/Color	OR and OD maps in V_1_ cross systematically approximately at right angle Most PWs in OR maps and about 50% of the blobs in V_1_ coincide with the center of OD domains The OR and color maps in V_1_ are each only loosely related to OD maps	(Bartfeld and Grinvald, 1992) (OI + ANAT) (Landisman and Ts'o, 2002) (OI + P)
OR/Color/Luminance	OR perception is influenced by both chromatic and luminance	(Clifford et al., 2003) (PSY)
OR/OD/SF	OR and OD maps in V_1_ often cross at right angles while most PWs are concentrated in the center regions of the OD columns albeit weaker than for OR/OD, geometric relationships are also observed between the OR and SF domains. The OD and SF maps in V_1_ are also spatially related: there is a tendency for the low SF domains to avoid the border regions of the OD columns.	(Hübener et al., 1997) (OI)
ON-OFF/RET/OR	The spatially separate ON and OFF subfields of simple cells in layer II-III of V_1_ exhibit topographically distinct relationships with RET and OR maps	(Lee et al., 2016) (2P)
OR/DIR/OD	OR, DIR, and OD maps in V_1_ are represented in the cortex in a mutually dependent manner Baskets cells in V_1_ establish connections with OR, DIR, and OD maps	(Kim et al., 1999) (OI) (Buzás et al., 2001) (OI + ANAT)
DIR/SF/Contrast	Perceived DIR is highly dependent on the SF and the contrast of the visual stimulus	(Lalanne and Lorenceau, 2006) (M)
SF/TF/Color	Cytochrome oxidase within blobs of V_1_ coincide with domains engaged in the processing of low SF and high TF contents of the visual scene	(Shoham et al., 1997) (OI)
RET/OR/Color	Apparent color-orientation bindings in the periphery can be influenced by feature binding in central vision	(Suzuki et al., 2013) (PSY)
OR/RET/SF	High SFs and low SFs activate OR maps in V_1_ when central and peripheral vision are respectively concerned	(Ribot et al., 2013) (OI + M)

*RF, receptive field; TF, temporal frequency. MEG, magneto-encephalography*.

Interactions and inter-dependency between all of the visual attributes. The question of the interactions and inter-dependences between all the attributes during visual perception is a key issue here since such interactions participate actively to the elaboration of the global visual perception. Let us examine this at the neuronal level first in V_1_ and then beyond, in superior visual areas.

#### V_1_ area

We have shown above that V_1_ contains two distinct populations of neurons: “Pop 1,” activated through the M and/or P channels, which is the largest and has the most convergence of attributes, and “Pop 2,” activated through the P and/or K channels, which is smaller and has less convergence (section Convergence at the Level of Single Neurons; Table 1). Pop 1 neurons (simple or complex) have rectangular receptive fields which are activated by orientation, velocity and/or direction of movement and are mostly binocular while Pop 2 includes neurons with circular receptive fields almost exclusively activated by colors through one eye. Correspondingly, we hypothesize that the degree of interactions and inter-dependence between the various visual attributes within each neuronal population corresponds to their degree of convergence. Thus, there would be more attribute interactions in Pop 1 than in Pop 2. But both neuronal populations are activated by the P channel, which suggests that attributes from each population may also interact and be inter-dependent. The fact that some neurons of Pop 2, located in the blobs in layer II-III, are also sensitive to velocity of movement supports this hypothesis (cf. Table 1). The next paragraph provides further supports for this.

Figure 5C shows that, viewed from the surface, V_1_ appears as a retinotopic map of visual space with “overlapping” functional spatially periodic maps that represent stimulus features such as edge orientation, direction of movement, spatial frequency etc. But, during visual perception, all these maps have to interact in a coordinated way. Their spatial relationships may ensure that all combinations of stimulus features are represented uniformly across the visual field. But the question here is “How?” Both experimental approaches and theoretical models attacked this question. From these studies, two main principles have emerged. First, *cortical maps in V1 are optimized for uniform coverage*. This principle permits continuous smooth mapping of stimulus properties across the cortical surface, and “coverage uniformity” that is uniform representation of combinations of map features over visual space (Swindale, 1991, 2000; Swindale et al., 2000). Here stimulus features such as edge orientation, velocity/direction of movement and spatial frequency are thought to be encoded in V_1_ by overlapping feature maps arranged so that the location of neurons activated by a particular combination of stimulus features can be predicted from the intersections of these maps. An alternative principle has been proposed stipulating that patterns of activity elicited by complex stimuli are best understood in the framework of a *single map of spatio-temporal energy* rather than by overlapping and intersecting multiple maps. The authors supporting such principle (e.g., Basole et al., 2003, 2006; Mante and Carandini, 2005) indeed observed that a single population of neurons can be effectively activated by *multiple* combinations of orientation, length, motion axis and speed.

#### Superior visual cortical areas

Considering the convergence of the three channels M, P, and K within both the dorsal and the ventral streams and the respective functional roles of each stream in visual processing, it is not surprising that multiple interactions occur between all the visual attributes within both streams (see Table 2 for details; cf. also section Evaluation of Convergence in Each Superior Visual Cortical Area).

Both streams are also linked through numerous reciprocal and unidirectional connections (e.g., Ungerleider et al., 2008; Pollen, 2011; see also Cloutman, 2013 for review). This enables additional interactions and inter-dependences. Functional data indeed support this notion. For example, such interactions may allow “integrating” simultaneously faces, houses, motion and action (Keizer et al., 2008). Such cooperation allows the perception of 3D object shape from 2D random-dot motion (Iwaki et al., 2013). It has also been demonstrated that a circumscribed damage to ventral stream impairs central motion perception, even for non-form motion (Gilaie-Dotan et al., 2013); cf. Table 5 for some more examples.

- Thus, all attributes of the visual scene interact and are inter-dependent during the elaboration of visual perception. This occurs in V_1_ and is further processed at the level of the superior visual cortical areas.

**Table 5:** Interactions between the dorsal and ventral streams, implicating interactions between all the attributes of the visual scene.

**Types of interactions**	**Summary of data**	**References**
Infero-temporal area (IT) ↔ Parietal Cx ↔ Pre-frontal Cx (associated with working memory)	Human – Mental rotation task – Manipulating figural complexity (simple vs. complex) to affect the figure recognition process (associated to ventral stream) + Manipulating the amount of rotation (0 deg vs. 90 deg) >> both dorsal and ventral streams are affected simultaneously by the manipulations + over-additive interaction + increased synchronization among multiple brain areas as task demand increases	(Koshino et al., 2005) (fMRI)
Dorsal stream ↔ Ventral stream	Interactions between both streams during adaptative behavior	(Goodale et al., 2005) – Review
Fusiform face area (FFA) ↔ Extra-striate body area (EBA) ↔ para-hippocampal place area (PPA) ↔ lateral occipital complex (LOC) ↔ MT/V5	Human – Using various stimuli: faces/objects, body parts – objects, scenes/objects, objects/scrambled objects and moving/stationary stimuli	(Spiridon et al., 2006) (fMRI)
**FF**: FST, LIP, TEO, TE and TF → → V4 **FB**: V4 → V2, V3 **FF and FB**: V4 ↔ V3A, MT, VIP, PIP, FEF	Monkey – Injections of tracers	(Ungerleider et al., 2008) (ANAT)
Dorsal stream ↔ Ventral stream	Humans simultaneously integrate images of faces, houses, motion and action. This is only possible through a binding process, with interactions between both the ventral and dorsal streams.	(Keizer et al., 2008) (PSY)
Dorsal stream ↔ Ventral stream	The V_1_/V_2_ complex and ventral cortical areas V_3_, V_4_ together with dorsal cortical areas LIP, VIP and 7a with additional contributions from motion areas MT/V5, FST and MST together with their sub-cortical relations have the physiological properties required to constitute a “posterior perceptual core” that underlies the normal primary perceptual experience of image content, space and sense of minimal self.	(Pollen, 2011) – Review
Dorsal stream ↔ Ventral stream	The perception of 3D object shape from 2D random-dot motion implicates cooperation between both the dorsal and ventral streams.	(Iwaki et al., 2013) (PSY)
Dorsal stream ↔ Ventral stream	Circumscribed damage to ventral visual cortex impairs central motion perception, even for non-form motion	(Gilaie-Dotan et al., 2013) (PSY)
Dorsal stream ↔ Ventral stream	Information is transferred directly between the ventral and dorsal streams at multiple stages and locations along their trajectories	(Cloutman, 2013) –Review
V4 + MT/V5	Color perception through S-cones implicates both the ventral and the dorsal streams. The combination of both signals would facilitate the extraction of shape-from-shadow signals to benefit global scene analysis and motion perception	(Conway, 2014) – Review
Dorsal stream ↔ Ventral stream	Dorsal and ventral attention systems use distinct circuits but they display collaborative roles	(Vossel et al., 2014) –Review
Dorsal stream ↔ Ventral stream	Posterior parietal cortex drives infero-temporal activations during 3D object vision	(Van Dromme et al., 2016) (fMRI)

*Cx, cortex; FF, feed-forward connections (symbol: → → ); FB, feed-back connections (symbol: → ); reciprocal connections indicated by the symbol “↔”; fMRI, Functional Magnetic Resonance Imaging. Other abbreviations, same as in other Tables*.

### Strabismic subjects *before* any medical intervention

Considering that the visual system sustains its general organization in spite of abnormal vision, the attributes of the visual scene also interact after infantile strabismus and are thus inter-dependent during the elaboration of visual perception. But, as a general rule, when brain processing and hence perception of one given visual attribute is altered, this has a negative impact on others. Such alteration might be even more extended than might be expected. Thus, for example, infantile strabismic amblyopes show localization deficits that are larger than expected when considering their losses in spatial resolution (Kiorpes et al., 1993). Also, thresholds for depth perception are increased when acuity is decreased (Husk et al., 2012); cf. Table 6 for more examples. Accordingly, the two general *principles of interaction and inter-dependence* also apply to cortical maps of subjects with infantile strabismus. Farley et al. (2007) even proposed that the global visual alteration resulting from infantile strabismus results in a “coordinated reorganization of multiple visual cortical maps.”

- The entirety of visual cortex follows both the *principle of convergence* and the *principle of interactions and of inter-dependencies* between the different attributes of the visual scene both in normally viewing and infantile strabismic subjects.
- Such principles may thus also apply in case of visual rehabilitation after infantile strabismus.

**Table 6:** Examples illustrating interactions between at least 2 visual attributes in adult mammals with infantile strabismus.

**Interactions**	**Main results**	**References**
Spatial location/acuity	Infantile strabismic amblyopes show localization deficits that are large relative to their losses in spatial resolution (in contrast with anisometropic amblyopes)	(Kiorpes et al., 1993) (PSY)
Contrast sensitivity/vernier acuity	When Vernier stimuli are equal in terms of effective contrast, the extent of the Vernier acuity deficit is reduced to approximately the extent of the spatial resolution deficit	(Kiorpes et al., 1993) (PSY)
OR / OD	In contrast to normally raised animals (cats), PW centers (in OR maps) after infantile strabismus no longer show consistent topographical relationship to the peaks of OD domains although OR and OD maps continue to cross orthogonally	(Engelmann et al., 2002) (OI)
SF/OR	After infantile strabismus, the OR map in V_1_ is altered with weakest (amblyopic) eye viewing only low SF	(Schmidt et al., 2004) (OI)
Acuity/depth perception	Depth thresholds are increased while acuity is decreased	(Husk et al., 2012) (PSY)

*OR, PW, SF, OD, OI, PSY, abbreviations same as in other Tables*.

*The latter is a major point in the present context since our main hypothesis here is that if improving the perception of at least one given attribute of the visual scene after early strabismus, after and even before eye surgery, it will improve the perception of some (if not all) other attributes. This hypothesis leads to interesting consequences, for example, that improving acuity through therapy will also be beneficial for spatial localization as well as the perception of movement etc… A few examples supporting this hypothesis will be evoked in section Data Which Support Our New Perspective*.

## Impact of the visual system on other sensory and motor systems

Many sensory and motor systems in higher mammals are strongly vision-dependent and are thus also affected in case of infantile strabismus. They are also of main interest here since rehabilitation of vision might also restore these systems and their associated functions. In other words, rehabilitation after infantile strabismus likely might be extended far beyond visual perception.

A good model to approach this issue is *postural stability*, which plays a major role for equilibrium and orientation of the body (e.g., Rougier and Lacour, 2006 for review). Of course, this very complex process requires various sensory-motor interactions, and depends on various skeletal segments and muscles whose actions are controlled by static and dynamic reflexes and dependent on attention. But postural stability is also strongly under the influence of visual, somato-sensory (including proprioception) and vestibular sensory systems as well as oculomotricity (e.g., Nashner, 1976; Horak, 2010; see also Paillard, 1971, 1976; Massion, 1994, 1997 for reviews).

*The impact of vision* on postural stability is rather well documented. The simplest evidence is that postural stability is better when both eyes are open than when they are closed (e.g., Baron, 1950; Gagey et al., 1973; Paulus et al., 1984; Isotalo et al., 2004; see also for example Guerraz and Bronstein, 2008 for review). Not surprisingly, blindness has dramatic consequences on posture (e.g., Alotaibi et al., 2016). This vision-dependence of posture has also been demonstrated in infantile strabismic subjects. As indicated in the Introduction, infantile strabismus also impairs postural stability (Marucchi, 1987; Marucchi and Gagey, 1987; Lions et al., 2014; Ezane et al., 2015). But of particular interest here the *quality of vision* in case of infantile strabismus (even before any surgery) has a substantial impact onto postural stability. Thus, Matsuo et al. (2006) reported that infantile strabismic children with some (even poor) binocular vision display better postural performance than those with none. Also, Gaertner et al. (2013) showed that bi-ocular visual stimulation improves control of posture for both near and far distances, in cases of convergent and divergent strabismus. Finally, even though vision is altered because of infantile strabismus, the postural parameters are also significantly better with the eyes open than when they are closed (Legrand et al., 2011).

*The impact* of the somato-sensory system *through extraocular proprioception* on postural stability is also well established in the literature. Recall that extraocular proprioceptive inputs originate from receptors within the extraocular muscles which are mainly located in the tendons and which project through the ophthalmic branch of the Vth nerve to multiple sites in the CNS (e.g., Milleret, 1988; Buisseret, 1995; Donaldson, 2000 for reviews). These receptors provide information about the position of the eyes to the brain during eye movements, including for example at the level of the vestibular nuclei (Ashton et al., 1988, 1989). Thus, under normal conditions, the extraocular proprioception contributes to maintain body equilibrium (Ushio et al., 1975, 1980; Eber et al., 1984; Roll et al., 1989). In infantile strabismus, the general idea is that such extraocular proprioception is unbalanced because of the misalignment of the eyes, thus contributing to generate postural instability (e.g., Legrand et al., 2011; Ezane et al., 2015; Bucci et al., 2016; Lions et al., 2016).

- *We propose here that any improvement of vision and/or extraocular proprioception after eye surgery (or any other appropriate therapeutic protocols) might also lead to functional improvement of vision-dependent systems including postural stability. Reshaping and re-equilibration of various neural networks would be required for this but brain plasticity is likely able to solve this problem whatever the age*.

## Data which support our new perspective

The three *principles of convergence, interaction and inter-dependency* underlie the processing of the various attributes in visual cortex during the elaboration of visual perception. Furthermore, the visual system influences other systems such as sensory and motor systems (cf. section Impact of the Visual System on Other Sensory and Motor Systems). *Based on these “rules” our hypothesis here is that the rehabilitation of perception after infantile strabismus should be substantially extended compared to presently. In fact, as developed below, this is already supported by some data in literature established by fundamental research*.

### Rehabilitation of visual perception

#### Present actions of ophthalmologists and collaborators in cases of infantile strabismus

In the clinic, the different forms of infantile strabismus are observed to affect both the sensory and the motor aspects of visual perception. It is also well known that infantile strabismus affects other abilities like postural stability (cf. section Impact of the Visual System on Other Sensory and Motor Systems). It has also been shown (in 55 children from 7 to 15 years old) that non-verbal abilities are altered in cases of infantile strabismus, with a significant relation between infantile strabismus and constructive praxia (*p* = 0.009), visual memory (*p* = 0.037), strategy formation (0.040) and the quality of drawings (Gligorović et al., 2011). Whether this is a direct consequence of infantile strabismus or whether the abnormal cerebral patterns (abnormal neuronal activities, asynchrony) which lead to infantile strabismus are the *primum movens* of both infantile strabismus AND other disabilities is presently unknown however.

Regardless of the answer, recall that ophthalmologists, orthoptists and optometrists presently focus the treatment of infantile strabismus on only three major items:

1. *Visual acuity*. The monocular strabismic amblyopia must be avoided and cured in all cases. This is performed classically through patching or through optical or pharmacological penalization of the fellow (non-deviated) eye to increase vision of the amblyopic (deviated) eye. The patching treatment is effective and sufficient in most cases. However, sometimes the effectiveness of these procedures may be limited because of poor compliance and variable outcomes. Additionally, if the amblyopia is severe, these treatments are difficult to initiate; amblyopia treatment can also be less effective if initiated after age 10. This partially explains the reason why additional “new” techniques such as “binocular therapy” have been proposed (cf. section Rehabilitation of Monocular Vision in Each Eye).
2. *Alignment of the eyes*. Eye alignment is adjusted through optical treatment and/or surgery. In some forms of infantile strabismus such as pure accommodative strabismus (which is a late-onset strabismus), only an optical treatment of hyperopia allows alignment of the eyes.
3. *Rehabilitation of binocular vision*. Rehabilitation can only be achieved in patients who do not display an *early-onset* infantile strabismus (i.e., developed before 12–24 post-natal months), and in patients for whom monocular amblyopia is avoided or cured AND alignment of the eyes is obtained.

Even focusing on these particular items, several challenges remain in the management of infantile strabismus. In addition to those evoked in the Introduction, two others exist. First, both amblyopia and infantile strabismus are linked since infantile strabismus may cause amblyopia, and also an amblyopic eye may become strabismic. However, so far, new therapeutic tools of treatment for visual rehabilitation mainly focus on the treatment of amblyopia in general and not on *strabismic* amblyopia only. Also, whether rehabilitation could be addressed to infantile strabismus without amblyopia remains unclear. This interdependency between amblyopia and strabismus along with the lack of effective new therapeutical tools are obstacles to reach a global rehabilitation of all visual and extra-visual aspects of infantile strabismus. Second, the mechanisms which lead to the development of amblyopia (including strabismic amblyopia) are presently rather well known, although still discussed (cf. for example Hess et al., 2009; Clavagnier et al., 2015). Due to these mechanisms, which implicate malleable neural networks, amblyopia may also be treated relatively easily through peripheral treatments (see above for details). In contrast, the mechanisms responsible for infantile strabismus and the lack of binocular vision remain less precisely identified. Most infantile strabismus at least seems however to result mostly from central and robust abnormalities which develop pre- or post-natally (Barnes et al., 2001; Bui Quoc and Milleret, 2014). It is therefore the cortical deficits which must be addressed. The same may apply to subjects with late infantile strabismus whose lack of binocular vision cannot be managed. But ophthalmologists, orthoptists and optometrists are still wondering how to treat the brain of infantile strabismic patients (Tychsen, 2005). Presently all treatments are systematically peripheral and, not surprisingly, are not always successful.

On the other hand, the rehabilitation of the other altered aspects of visual perception, concerning spatial location, contrast sensitivity, motion or orientation detection and color vision has not been considered to date and therefore must be developed. In particular, the abnormal perception of the deviated eye (even after realignment) must be restored (Economides et al., 2012). This also applies to the likely abnormal perception deriving from the non-deviated eye (see above). However, medical and paramedical professionals currently lack tools to both evaluate and rehabilitate these aspects of visual perception. Hopefully, the hypothesis developed here will inspire improvements for this.

#### Data showing that rehabilitation of vision may be improved after infantile strabismus

Some data in the literature already support the hypothesis we are developing here. Of particular interest, they have been obtained by using only *non-invasive innovative techniques*.

##### Rehabilitation of monocular vision in each eye

As underlined above, preventing and/or treating amblyopia of the deviated eye is the first condition to rehabilitate vision in the case of infantile strabismus. Monocular equilibrium and maximal vision in both eyes are *absolutely* necessary to then have good chances to obtain normal binocular vision (whether or not eye surgery is involved). Thus current efficient techniques to penalize the good eye forcing the amblyopic eye to improve *are imperative*. On the basis of the present knowledge, the efficiency of this technique is acknowledged, even if each eye works separately. Indeed, for a short period of time, penalization/patching may be permanent until obtaining iso-acuity. But it may then be conducted part-time, which does NOT constantly make each eye work separately.

However, it may be of interest *to complement the patching method by* a *binocular therapy* to eliminate amblyopia and promote cooperation between the two eyes. R. Hess, B. Thompson and their collaborators are developing precisely such an approach and are among the leaders in the field (e.g., Baker et al., 2007; Mansouri et al., 2008; Hess et al., 2010a,b, 2011; To et al., 2011; Black et al., 2012; Zhou et al., 2012; Li et al., 2013, 2015; see also Hess and Thompson, 2013, 2015; Hess et al., 2014a,b for reviews). In brief, the method is based on a dichoptic stimulation which aims to increase acuity and decrease the interocular suppression phenomenon in amblyopia. *Of particular interest here, to reach equal stimulation, the contrast of the image presented to the amblyopic eye is increased while the contrast of the image presented to the fellow fixing eye is decreased*. We must note however that nowadays this technique has mainly proven effective in adults because testing on children is much more difficult to access. Whether it is applicable to all types of strabismus also remains unclear. Presently at least, binocular therapy thus cannot be considered as an *alternate* method to replace patching or penalization.

Also of interest, quite recently, Marc Bear and his colleagues have proposed a novel and promising technique to restore acuity which is completely different from the above described ones. They indeed hypothesized that “a period of retinal inactivity can reduce the threshold for synaptic potentiation such that subsequent visual experience promotes synaptic strengthening and increased responsiveness in the visual cortex” (Fong et al., 2016). To test this hypothesis, they used two different animal models. In young mice, they have shown that the otherwise stable loss of cortical responsiveness caused by monocular deprivation is reversed when binocular visual experience follows temporary inactivation of the retinas (through binocular intra-vitreal tetrodotoxin [TTX] injection). In 3 month-old kittens, they have shown that a severe impairment of visual acuity is also fully reversed by binocular experience following treatment (again binocular intra-vitreal TTX injection) and, further, that prolonged retinal inactivation alone can erase anatomical consequences of monocular deprivation. They conclude that temporary retinal inactivation represents a highly efficacious means to promote recovery of function. It indeed reveals to be able to “reboot” the brain, which allows the lazy (amblyopic) eye to recover acuity permanently, without any detriment to the strongest (fixing) eye. Until now, such a technique has only been applied to animals with amblyopia. The authors now plan to determine whether the treatment might also be suitable for clinical use in humans.

- Although not included yet in the current treatments of amblyopia after infantile strabismus by medical and paramedical professionals, at least the data obtained by Hess and his group obtained through the “binocular therapy” strengthen the hypothesis we have developed here since modifying the contrast of patterns presented to each eye may have a positive impact on the perception of the high spatial frequencies, i.e., acuity.

##### Rehabilitation of binocular vision and 3D perception

Binocular vision is also abnormal in the case of infantile strabismus and/or amblyopia (strabismic amblyopia). Several protocols have been developed to restore it and some are also based on the principles developed above (section Principle of Convergence in Visual Cortex and Principle of Interactions and of Inter-Dependency of all the Attributes of the Visual Scene).

As previously discussed, balanced monocular vision in both eyes is necessary for restoring binocular vision, in combination with the alignment of the eyes. Dichoptic stimulation has been used in the twentieth century for the treatment of infantile strabismus and amblyopia, and orthoptic treatment of abnormal retinal correspondence has attempted to reestablish binocularity. Unfortunately, this has been proven ineffective with the additional risk of creating a permanent diplopia. For these reasons, although interesting, innovative and promising, dichoptic vision training such as that proposed by R. Hess and B. Thompson' work must be considered with caution even if such training rehabilitates 3D perception in some cases.

Brain stimulation is another option. It aims at both re-adjusting the “excitatory/inhibitory balance” and re-equilibrating the “cortical synchronization” (Deco et al., 2014) which are both altered in infantile strabismus (cf. above). Of interest here, transcranial direct current stimulation of the brain has also been recently shown to target specific processing channels in human visual cortex (Costa et al., 2015). This method is already currently used in clinics in cases of brain disorders such as epilepsy, bipolarity, Parkinson's disease and schizophrenia (e.g., Koch, 2013; Kimiskidis et al., 2014; for reviews). On such bases, Benjamin Thompson, Robert Hess and their collaborators have compared the effect of dichoptic treatment alone and in combination with visual cortex trans-cranial direct current stimulation on measurements of binocular (stereopsis) and monocular (visual acuity) visual function in a group of 16 young adults (mean age 22.1 ± 1.1 years) with amblyopia. It was shown that the dual treatment leads to greater improvements in stereoacuity than dichoptic treatment alone (e.g., Thompson et al., 2010; Clavagnier et al., 2013; Spiegel et al., 2013b).

*Another approach is the use of motion stimulation which can enhance stereopsis*. In 120 normal children and 30 strabismic patients (9 esotropia, 14 exotropia, 7 intermittent exotropia), Handa et al. (2010) studied the binocular performance with static or moving stereograms. In exotropic patients, they found that 19 subjects (90.4%) succeeded in performing the moving stereogram test whereas 13 (61.9%) succeeded in only performing the static stereogram test, suggesting a possible effect of motion stimulation in improving stereopsis.

- Equalizing acuity (or almost so) in both eyes increases stereo-acuity and thus binocular vision. The use of moving stimuli also improves stereopsis and therefore binocularity. These examples again support our hypothesis.

##### Rehabilitation of contrast sensitivity

Contrast sensitivity may also be improved through dichoptic training in case of amblyopia. Quite recently, Li et al. (2015) demonstrated this in 30 adults after 10 days of dichoptic training delivered through a dichoptic video game viewed through video goggles (*n* = 15) or an iPod touch equipped with a lenticular overlay screen (*n* = 15). It should be noted that similar improvement has been observed with the two methods for all spatial frequencies which were tested.

Comparing contrast sensitivity before and after anodal trans-cranial direct current stimulation in 8 of 13 amblyopic adults, Spiegel and colleagues showed that contrast sensitivity may also be transiently improved using such an approach (Spiegel et al., 2013a,b). In the same study, the authors also demonstrated that the activation of the visual cortex itself may be normalized in individuals with amblyopia after this type of stimulation.

Finally, video game training itself (without any specific dichoptic device) has also been shown to enhance contrast sensitivity in case of amblyopia (Li et al., 2009).

- Consistent with our *principles of interaction and of inter-dependency*, these results demonstrate that at least acuity and contrast sensitivity are tightly inter-linked: increased acuity may increase contrast sensitivity and *vice versa*.
- The data presented above also show that these types of interactions apply to a large range of spatial frequencies, which is, of course, beneficial for visual perception in the present context.
- Increasing synchrony within the visual cortex, by functionally influencing the neuronal networks underlying the treatment of information in relation to all the visual attributes, may contribute to the rehabilitation of contrast sensitivity.

Note that the data summarized above have been obtained from a wide range of ages: children from ~5 years and adults including elderly subjects, with however a predominance of adults. This allows underlying the existence of brain plasticity late in life (e.g., Mansouri et al., 2014) which likely results from the modulation of the inhibitory intra-cortical networks (Milleret and Buser, 1984, 1993; Watroba et al., 2001; Baroncelli et al., 2011). Once again, this is fundamental in the present context of rehabilitation since our final goal is to extend rehabilitation of vision after infantile strabismus as much as possible, i.e., in terms of both perception and age.

##### Motion detection and orientation assessment

Amblyopic subjects, whether strabismic or not, have also been shown to exhibit a global deficit in motion detection in both eyes. Thus, for example, Davis et al. (2008) reported that the peaks of motion-onset visually evoked potentials (VEPs) observed after visual stimulation of the amblyopic (strabismic) eye occur later than the ones observed from the fellow (non-strabismic) eye in visual cortex of adult patients with early- or late-onset strabismic amblyopia. These same authors also found that the peak times of VEPs observed after visual stimulation of the amblyopic and the fellow eyes are shorter than normal. Accordingly, Thompson et al. (2008) reported that the perception of moving patterns is abnormal in both eyes. Abnormal cortical processing of pattern motion in areas MT and MST of the dorsal stream has also been shown with functional magnetic resonance imaging (Thompson et al., 2012). Moreover, using a psychophysical approach, Husk et al. (2012) showed that structure-from-motion processing, which requires cooperation between both the dorsal and ventral streams (see Table 2) is altered. The efficiency in processing local orientation (implicating the ventral stream, where orientation coherence is processed) has also been reported to be poorer than normal (Husk and Hess, 2013). But as far as we are aware, no study has yet proposed any new therapeutic techniques that would focus on the improvement of these altered components in cases of infantile strabismus or amblyopia.

##### Color vision

In patients with late- but not early-onset strabismic amblyopia, Davis et al. (2008) have also reported that the peak times for color VEPs are significantly longer than normal when the amblyopic eye is stimulated. However, as with motion and orientation detection, we are not aware of any study that has ever examined any possible improvements in color vision after treatment of infantile strabismus. Not any improvement of strabismus using rehabilitation techniques focusing on color vision has been reported either.

### Rehabilitation of posture *after strabismus surgery* through visual perception

In section Impact of the Visual System on Other Sensory and Motor Systems, we discussed how the *quality of vision* of infantile strabismic children positively influences postural stability before any medical intervention, even if this vision is poorer than normal. We also discussed that oculomotricity contributes actively to posture at least through extraocular proprioception.

In addition, it has been established recently that: (a) postural control of children with infantile strabismus may be improved progressively within the weeks (2–8) following eye surgery (Legrand et al., 2011); (b) wearing prisms instead of having eye surgery alters rather than improves postural stability (Legrand et al., 2012). For these authors, the new “relations” that exist between the extraocular muscles and the CNS are thus of vital importance in the process of improving posture after eye surgery; (c) the quality of visual inputs, in terms of acuity and binocular vision at least, is of great importance in improving postural stability after infantile strabismus surgery (Lions et al., 2016); (d) The improvement of extraocular proprioceptive information which results from eye surgery also plays a major role in improving postural control (Bucci et al., 2016). The latter observation is directly linked to the improvement of the *quality of eye movements* after eye surgery. It has been demonstrated that the speed and accuracy of saccades, the vergence and the combined eye movements (and therefore binocular coordination) are generally poor before eye surgery and much better after eye surgery (Bucci et al., 2002, 2009).

- From this example, in agreement with our hypothesis, we demonstrate that rehabilitation after surgery for infantile strabismus may also take place in domains other than acuity, ocular alignment and binocularity (thus stereopsis), and even far beyond the visual system itself. Postural stability indeed involves very complex sensory-motor networks extending from the head to the feet and *vice versa*.
- Some questions however remain such as: (i) Does the rehabilitation of postural components by strabismus eye surgery leads to rehabilitation to normal performance levels? (ii) If yes, how long would such rehabilitation take? (iii) Are performance levels sustained over time? (iv) How much postural rehabilitation may be expected after treatment of different types of strabismus? etc… These questions are presently under study.
- With more knowledge and by installing adequate infrastructure (for both specific medical follow-up and training), our view is that, in the near future, it might become possible to optimize the rehabilitation of many children with infantile strabismus, far beyond visual perception, including postural stability. This could occur after eye surgery but we suggest that such improvements could be achieved by combining surgery with other methods, including daily visual experience and perhaps specific visual trainings (see below). We also believe that such rehabilitation might also be extended to adult subjects.

## Conclusion

In cases of infantile strabismus (for recall occurring during childhood), with either early-onset or late-onset, ophthalmologists, orthoptists and optometrists presently aim at rehabilitating vision by focusing on monocular visual acuity (prevention and treatment of amblyopia), motor balance/symmetry/alignment of the eyes, extraocular proprioceptive balance/symmetry in both eyes and binocularity/stereopsis. The perceptual alterations of the other attributes of the visual scene such as spatial location, orientation, velocity/direction of movement, contrast and color are therefore not treated. Yet such attributes play a major role during visual perception. The alterations of vision-dependent systems which, for example, lead to the alteration of postural stability, are also not explicitly taken into account. This would be useful though since, among other functions, postural stability affects locomotion and orientation of body in space. Medical and paramedical practitioners are aware of this problem. However, as outlined above, appropriate tools do not currently exist to address these issues. In addition, the incomplete knowledge of the complex relationships between strabismus and the brain is an impediment. Here, to deal with this problem, we propose that rehabilitation of perception after infantile strabismus may be extended from present practices, possibly with relative ease and/or by using non-invasive approaches.

Based on the present knowledge of the organization of the visual system in higher mammals including humans, our first proposal here is that the current *visual rehabilitation* might be extended to ALL (or almost all) of the attributes of the visual scene which are altered in case of infantile strabismus. The information related to all these attributes *converge, interact* and are *inter-dependent* with numerous common targets within the visual system, in particular at the level of the visual cortex, from V_1_ and beyond, from individual neurons to complex neuronal visual networks. *If the perception of one given visual attribute is improved, for example high spatial frequencies (i.e., acuity), we would expect that this might also improve the perception of other (if not all) visual attributes*. For instance, this would mean that the practice of addressing only high spatial frequencies, as is currently the case in the treatment of monocular amblyopia, could in itself improve the perception of the other visual attributes in cases of amblyopia and/or strabismus. Two neuronal populations are however distinguishable in V_1_, namely the populations Pop _1_ and Pop _2_, which differ both by their size and their amount of convergence (cf. section Convergence at the Level of Single Neurons and Table 1). Since the population Pop _2_ has the smallest size and the less convergence, we therefore expect that impairments in color perception after infantile strabismus may be more difficult to rehabilitate through convergence and interaction processes than the perception of the other attributes. However, interactions between the two populations Pop 1 and Pop 2 in V_1_ and visual processing in superior visual areas could compensate for this (cf. Table 2).

As developed in section Data Which Support Our New Perspective, our hypothesis is already supported by published data. These results could have impact for future. However, only four attributes of the visual scene are presently really considered: the classical ones (visual acuity, i.e., spatial frequency, binocularity, i.e., eye alignment and stereopsis, i.e., 3D perception) and also the contrast. We hypothesize that other (if not all) attributes may also be involved, i.e., may improve by themselves but it is not currently known. Therefore, firstly, it would be of interest to examine this with appropriate psychophysical tests. For example, it would be informative to establish that the perception of velocity and direction of movement have also been improved in each situation described in the previous paragraph after rehabilitation of infantile amblyopia/strabismus. If not, developing specific tools to rehabilitate and/or stimulate perception of movement/direction could lead to improve global visual perception in cases of infantile strabismus. Improving spatial location could also be beneficial. New and appropriate perceptual training programs would also need to be developed. This is also relevant for the other visual attributes. This would be challenging but, if the results are as we expect, this may have great interest for patients with infantile strabismus, whatever their age.

Furthermore, we have shown that postural stability may also be improved when visual perception is improved after infantile strabismus. This indicates that the functionality of other systems which differ from the visual one but which are vision-dependent may also be improved when visual perception is improved. Promising data and the development of new therapeutic strategies already favor such new perspectives (cf. section Data Which Support Our New Perspective).

Note that: (a) our hypothesis applies to infantile strabismus with either early-onset (developing within the first 24 months of birth) or late-onset, with the limitation that rehabilitation of binocular vision and thus of 3D perception may not be expected in the former case; (b) in principle, our hypothesis may apply to all forms of infantile strabismus (convergent, divergent …); (c) the simultaneous rehabilitation of different visual attributes has been already obtained both in children and adults with infantile strabismus. This is consistent with our hypothesis which considers that the visual system is essentially organized the same way in both cases. This is also consistent with brain capacity to “learn” throughout life including in adulthood (e.g., Buonomano and Merzenich, 1998; Gilbert et al., 2001, 2009; Sales et al., 2011), and even has the capacity of modifying the functional specializations of visual cortical areas (Adab et al., 2014; Chen et al., 2016). However, our expectation is that rehabilitation may be faster and/or even more extensive in youngest subjects because of a higher plasticity, in particular during the global critical period; (d) each visual attribute has its own critical period with its own time course (cf. Introduction and section Principle of Convergence in Visual Cortex). Thus, to optimize the rehabilitative processes discussed here, it would seem pertinent to, when possible, take advantage of the optimal plasticity that exists during each critical period, for each visual attribute. Unfortunately, this is presently difficult given the time course of the critical period is known for only two visual attributes (*binocular vision* and *acuity*); (e) rodent models have allowed identifying neuro-chemical changes that control the onset of the critical period for binocular vision, as well as molecular and structural brakes that lead to the diminution of the plasticity thereafter (e.g., Hensch, 2005; Sugiyama et al., 2008, 2009; Hensch and Bilimoria, 2012; Prochiantz et al., 2014; Prochiantz and Di Nardo, 2015; Bernard and Prochiantz, 2016 for reviews). However, no application of these findings has been developed for humans yet, as far as we are aware. Any application would likely help in the present context, although the functioning of the brain is probably too complex to be adjusted simply by a drug (or a cocktail of drugs). In addition, manipulating the brain of a child during development raises ethical questions that would need to be addressed; (f) in all cases, the eyes need to be aligned (or realigned) to ensure the success of the rehabilitation process of perception and to extend this, not only for binocular vision but also for eye movements themselves. We reiterate here that if eye movements are (still) abnormal (in case of strabismus; before and/or after treatment), visual perception and all other processes which are vision-dependent will remain altered; (g) in rehabilitation of vision, all the visual cortical areas from V_1_ and beyond need to be rehabilitated, with recognition that the former will need to be rehabilitated first (e.g., Hooks and Chan, 2007); (h) exercise and attention play major roles during the rehabilitation processes we have discussed, up to adulthood. For example, it has been demonstrated recently that voluntary physical exercise may promote ocular dominance plasticity in V_1_ of the adult mouse (Kalogeraki et al., 2014). It has also been shown that attention may enhance spatial resolution (Mineault et al., 2016; see also Barbot, 2016 for review); (i) during the perceptual learning training, the stimuli being used, the duration of each session, the frequency of the sessions and their total duration are of importance and need to be determined carefully. These parameters may be subject to change depending on the attributes concerned; (j) perceptive rehabilitation after the training sessions seems to have a long-term effect, through a meta-plasticity of the brain, which is evidently fundamental here (e.g., Bocci et al., 2014).

To conclude, we propose approaches to improve rehabilitation after infantile strabismus and some suggestions for their implementation. Some data from fundamental research already support these views. However, further work remains to be carried out. To proceed, tools already in existence and those under development will need to be validated in order to be systematically available for medical practitioners. A multidisciplinary approach will also be required. A deeper cooperation between medical and paramedical practitioners from different domains will be essential. The body is indeed a “multi-sensorial” and a “sensory-motor” system and not a “mono-sensorial” one. A more profound working relationship between practitioners and neuroscientists will also be essential to allow a rapid transfer of fundamental knowledge to practice. The end result will be of great benefit to patients.

## Author contributions

Study concept and design: CM. Neural bases for visual perception, psychophysical and modeling data about vision and postural stability (normal condition and after strabismus): CM. Epidemiology, etiology and treatments of strabismus: EB. Critical revision of the manuscript: CM and EB.

### Conflict of interest statement

The authors declare that the research was conducted in the absence of any commercial or financial relationships that could be construed as a potential conflict of interest.
